# Diversity and evolution analysis of RNA viruses in three wheat aphid species

**DOI:** 10.1186/s12864-025-11512-1

**Published:** 2025-04-07

**Authors:** Ke-Hui Feng, Yu-Hua Qi, Zhuang-Xin Ye, Ting Li, Gao-Yang Jiao, Chuan-Xi Zhang, Jian-Ping Chen, Gang Lu, Jun-Min Li

**Affiliations:** 1 State Key Laboratory for Quality and Safety of Agro-Products, Key Laboratory of Biotechnology in Plant Protection of MARA, Zhejiang Key Laboratory of Green Plant Protection, Institute of Plant Virology, Ningbo University, Ningbo, China; 2 College of Forestry, Nanjing Forestry University, Nanjing, China

**Keywords:** RNA virome, Insect-specific viruses, Endogenous viral elements, Wheat aphids

## Abstract

**Background:**

Although advances in metagenomics, viral diversity and non-retroviral endogenous viral elements (EVEs) in wheat aphids remain underexplored. By analyzing 470 publicly available datasets and one laboratory-generated transcriptome, the RNA virome and EVEs in the genomes of *Sitobion avenae*, *Schizaphis graminum*, and *Rhopalosiphum padi* were systematically investigated.

**Results:**

We identified 43 RNA viruses, including 12 novel and 31 known RNA viruses. These viruses were widely distributed and abundant in different geographic populations of three wheat aphid species. +ssRNA viruses were the dominant type of aphid viruses. Besides, 90 EVEs were discovered in the genomes of three aphid species. In addition, the EVEs exhibit potential domestication and novel functional roles within aphid genomes.

**Conclusions:**

This study expands the understanding of RNA virus diversity in aphids and provides valuable insights into the potential functions of EVEs in virus-host coevolution.

**Supplementary information:**

The online version contains supplementary material available at 10.1186/s12864-025-11512-1.

## Introduction

Wheat (*Triticum aestivum*) is one of the important staple food crops worldwide, playing a critical role in global food security. However, wheat aphids, including *Sitobion avenae* (Fabricius), *Schizaphis graminum* (Rondani), and *Rhopalosiphum padi* (Linnaeus), pose a major threat to wheat production [1]. In 2023, *S. avenae*, *S. graminum* and *R. padi* were classified as major crop pests in China [2, 3]. These aphids not only cause direct damage to crops but also transmit several plant viruses, such as barley yellow dwarf virus and cereal yellow dwarf virus [4–6].

In addition to transmitting plant viruses, aphids also harbor numerous insect-specific viruses (ISVs). For example, the Aphid lethal paralysis virus has been identified in various aphid species and is known to affect the movement and lifespan of *R. padi* [7–9]. Besides, the Rhopalosiphum padi virus has been found to reduce the reproductive capacity of aphid host [10]. Other ISVs, such as the Brevicoryne brassicae virus, the rosy apple aphid virus, and the Acyrthosiphon pisum virus, demonstrate the remarkable viral diversity among aphid species [11–13]. In recent years, advances in next-generation sequencing (NGS) technology and bioinformatics tools have facilitated the discovery of numerous novel aphid-associated RNA viruses [14]. For instance, virus-like sequences related to nege-, kita-, flavi-, tombus-, phenui-, mononega-, narna-, chryso-, partiti-, and luteoviruses have been identified in *S. avenae* and *R. padi* collected from wheat fields in Japan [15]. Moreover, an RNA virome study identified 18 bunyaviruses from 10 aphid species, and aphid bunyavirus 1 (ABV-1) can be transmitted horizontally among aphids through plant feeding and vertically through reproduction [16]. A recent study showed that plants modulate aphid honeydew secretion and affect the horizontal transmission of densovirus in *Myzus persicae* [17]. Besides, a novel aphid RNA ISV has also been reported to affect the stylet penetration activity of *Aphis citricidus* and facilitate horizontal viral transmission [18]. However, the viral diversity in wheat aphids and whether these viruses participate in gene expression in aphid hosts during long-term evolution remain unknown.

During long-term host-virus co-evolution, viral sequences can integrate into host genomes, giving rise to the emergence of endogenous viral elements (EVEs) and contributing to the genetic diversity of their hosts [19, 20]. EVEs are originally derived from viral RNA or DNA and are ubiquitous in the genomes of various organisms, including fungi, plants, and animals [19]. They have been increasingly reported in the genomes of arthropod vectors, such as aphids and rice planthoppers. The non-retroviral EVEs have been firstly reported in *Aphis glycines*, and many are transcriptionally active [21]. 10 EVEs were found in six aphid genomes and provide evidence of over 60 million years of virus-host co-evolution [22]. In addition, The EVEs derived from invertebrate iridescent virus 6 of the genus Iridovirus were found in the genomes of three rice planthopper species, and these EVEs were detected in the transcriptomes of these three planthopper species [23]. Huang et al. discovered that endogenous toti-like viral elements (ToEVEs) are ubiquitously integrated into the genomes of three planthopper species. Notably, one ToEVE in *Nilaparvata lugens*, has been co-opted by its host and plays essential roles in planthopper development and fecundity [24].

In this study, we performed a comprehensive analysis of the RNA viromes of three wheat aphids (*S. avenae*, *S. graminum*, and *R. padi*) using publicly available datasets and transcriptomes from field samples. Additionally, we analyzed the genomic sequences of three wheat aphids to identify potential EVEs. By expanding the catalog of wheat aphid-associated RNA viruses and characterizing their EVEs, our work provides new insights into the diversity and evolution of insect viromes. These findings not only contribute to our understanding of the interplay between viruses and their hosts but also provide a theoretical foundation for future studies on the functional roles of EVEs in aphid biology and their implications for the transmission of plant viruses.

## Materials and methods

### Aphid sample collection and maintenance

The *S. avenae*, *S. graminum* and *R. padi* strains were collected in a wheat field in Jinan, China. The three wheat aphid strains were maintained separately on wheat plants at 26 °C ± 1 °C, with a photoperiod of 16 h light: 8 hdarkness and a relative humidity of 70%±10%.

### RNA sequencing (RNA-seq) libraries of wheat aphids from the public database

About 115 RNA-seq datasets of *S. avenae*, 182 RNA-seq datasets of *S. graminum* and 173 RNA-seq datasets of *R. padi* were retrieved from the NCBI SRA repository. The datasets were filtered based on the following criteria: Firstly, at least one dataset submitted by each unit was selected, as the virus compositions may vary across different submissions; Secondly, when several biological replicates were available, the dataset with the largest total number of bases was selected; Thirdly, datasets were only used for further bioinformatics analysis if they contained viruses. Abbreviations and detailed information of these wheat aphid datasets are provided in Table S1. The species identification of *S. avenae*, *S. graminum* and *R. padi* were confirmed by amplifying, cloning, and validating the mitochondrial cytochrome oxidase I (COI) gene through Sanger sequencing using universal primers. The primers used for species identification are listed in Table S2.

### RNA-seq libraries of *S. avenae* generated from field samples

The aphid samples (*S. avenae*) were collected from Ningbo, China in 2022. Total RNA was extracted from approximately 15–20 aphids using TRIzol reagent (Invitrogen, Carlsbad, CA, USA). The extracted RNA was used to prepare a library for Illumina high-throughput sequencing. Paired-end (150 bp) sequencing was conducted on the Illumina HiSeq 4000 platform (Illumina, CA, USA) by Novogene (Tianjin, China). The transcriptome raw reads were deposited in SRA under accession number SRR30230442.

### Determination of the genome ends of the ISV identified from *S. avenae* sample

The complete genome sequence of the Sitobion avenae iflavirus 1 (SaIfV1) identified from field sample was determined through rapid amplification of cDNA ends (RACE). The cDNAs were generated using SMARTer^®^ RACE 5′/3′ kit and amplified by PCR using gene-specific primers (GSPs) and a universal primer mixture (UPM). The PCR products were then cloned into 5× TA/Blunt-Zero Cloning (Vazyme, Nanjing, China), and subsequently Sanger sequencing. Detailed information on the primers used for RACE and viral genome verification are provided in Table S2.

### Dataset reassembly and RNA virome discovery

Quality assessments of sequencing reads from 123 selected transcriptome datasets in the SRA repository, along with the transcriptome of the field sample, were conducted using FastQC and Trimmomatic. Filtered reads were reassembled *de novo* using the two assembly software packages, Trinity and metaSPAdes, with default parameters [25, 26]. The assembled contigs were compared with the NCBI viral RefSeq database using Diamond BLASTx [27]. Strict criteria were applied to identify putative novel viruses in each dataset. Firstly, Diamond BLASTx was set with an E-value cutoff of 1 × 10^− 20^. Secondly, viral homology contigs were required to meet minimal coverage and length criterion of 20× and 500 bp, respectively. Additionally, these contigs contained complete open reading frames (ORFs) of predicted viral RNA-dependent RNA polymerase (RdRP). Finally, regions of virus-like contigs matching the reference viruses were extracted and compared with the NCBI nucleotide and non-redundant protein databases to eliminate false positives. The virus-like sequences identified in three wheat aphids was confirmed by RT-PCR, followed by Sanger sequencing (primers are listed in Table S2).

### Virus genome annotation and phylogenetic analysis

The newly identified viral contigs were annotated with InterPro [28]. The RNA-dependent RNA polymerase (RdRP) regions of the newly identified viruses, together with RdRP sequences of reference viruses, were used for phylogenetic analysis. The RdRP sequences were aligned with MAFFT [29], and ambiguously aligned regions were trimmed by Gblock [30]. The best-fit model of amino acid substitution was evaluated by ModelTest-NG [31]. Maximum likelihood (ML) trees were constructed using RAxML-NG with 1000 bootstrap replications [32]. Details of all the reference sequences used in the phylogenetic analysis are listed in Table S4.

### Relative abundance of the ISVs in wheat aphid dataset

To identify ISV-derived viral reads, raw reads from each wheat aphid dataset were aligned to the corresponding ISV contigs using Bowtie2 software [33]. To explore the relative abundance of the newly identified ISVs across the different wheat aphid datasets, unassembled transcriptome reads of each dataset were mapped back to the corresponding viral contigs. A total of 161 representative datasets (65 *S. avenae*, 58 *S. graminum*, and 38 *R. padi*) were selected based on the following criteria: (1) data size over 1 Gb; (2) removal of biological replicates, retaining the dataset with the largest total number of bases. Detailed information on these datasets is provided in Table S1. The relative abundance of ISVs in each wheat aphid dataset was calculated and normalized using transcripts per million (TPM) values, as follows: $\:{a}_{j}$=$\:\frac{{b}_{j}/{c}_{j}}{{\sum\:}_{j=1}^{n}{b}_{j}/{c}_{j}}\times\:{10}^{6}$. In this equation, $\:{a}_{j}\:$represents the TPM of viral contig j, $\:{b}_{j}\:$represents the number of uniquely mapped fragments in a dataset, $\:{c}_{j\:}$represents the length of viral contig j, and n is the total number of viral contigs [34, 35]. The normalized relative abundance of ISVs in each dataset was further analyzed to PCA using TBtools V2.086.

### Small RNA sequencing and analysis

The cDNA libraries of *S. avenae* collected from the field were prepared using the Illumina TruSeq Small RNA Sample Preparation Kit (Illumina, CA, USA), and sRNA sequencing was performed on an Illumina HiSeq 2500 by Novogene (Tianjin, China). The raw reads of sRNAs were quality-controlled to remove adapter, low-quality, and junk sequences, and clean sRNA reads with lengths of 18–30 nt were extracted with FASTX-Toolkit v0.0.14 (http://hannonlab.cshl.edu/fastx_toolkit) and mapped to the identified viral contigs using Bowtie software with a perfect match (i.e., allowing zero mismatches) [36]. Downstream analyses were performed using custom Perl scripts and Linux shell bash scripts.

### Discovery and analysis of EVEs in the genomes of wheat aphids

The scaffold-level and chromosome-level genome assemblies of three wheat aphid species (*S. avenae*, *S. graminum*, and *R. padi*), were retrieved from the NCBI genome database with the accession numbers GCA_019425605.1, GCA_020882235.1, and GCA_020882245.1, respectively. Protein sequences of all identified wheat aphid viruses were searched against the genomes of the wheat aphid species using the tBLASTn algorithm with a cutoff E-value ≤ 10^–9^. The potential EVEs were then extracted from the genomes accordingly and used to search against entire protein database of NCBI to eliminate false positive hits. R (4.2.3) was used to compare the distribution of EVEs in three wheat aphid species. Additionally, to investigate sequence identities among the identified wheat aphid EVEs, alignment of viral RdRP-derived EVEs was performed using BioEdit Sequence Alignment Editor (version 7.1.11) and distance matrix analysis of EVE amino acid sequences was performed with MegAlign program (version 7.1.0) [37, 38]. The identified EVE sequences from wheat aphids are provided in fasta format as provided in Source Data. The presence of the discovered EVEs in the genomes of the three wheat aphid species was confirmed by PCR followed by Sanger sequencing.

### Transcription profiles of EVEs in publicly available wheat aphids

Public RNA-seq datasets of three wheat aphids were retrieved and analyzed from the NCBI SRA repository to explore the potential transcripts of the wheat aphid EVEs. A total of 161 representative datasets were selected (65 *S. avenae*, 58 *S. graminum* and 38 *R. padi*) (Table S1). The quality-trimmed raw reads of each dataset were mapped to the identified EVEs in the corresponding wheat aphid species using Bowtie2 (v2.3.5.186). The relative abundance of EVEs in each sample was normalized as fragments per kilobase of transcript per million mapped reads (FPKM) and average counts were used to quantification.

### Analysis of potential EVE transcripts in genomes of the three wheat aphids

To identify transcripts containing EVEs in wheat aphid species, raw reads from publicly available datasets were *de novo* reassembled using Trinity and metaSPAdes software [21, 25, 26]. The assembled contigs were then searched against a local customized database, which comprised all identified wheat aphid EVEs. To confirm the location of EVE transcripts within the wheat aphid genomes, the sequences of the identified EVE transcripts were extracted from the wheat aphid transcriptomes and used as a query to search against the corresponding genomes of wheat aphid. The matched region of EVEs in wheat aphid genomes was retrieved to predict open reading frames (ORFs) using the online ORF Finder server (https://www.ncbi.nlm.nih.gov/orffinder). The abundance of EVEs was measured by realigning the quality-controlled transcriptome raw reads back to the EVE transcripts. The selected EVE transcripts in wheat aphids were verified by RT-PCR, followed by Sanger sequencing.

### sRNA profiles of wheat aphid EVE transcripts

To investigate the sRNAs derived from EVEs, the publicly accessible small RNA datasets from NCBI SRA were selected. Detailed information on these wheat aphid datasets is provided in Table S1. The methods used were the same as those for small RNA analysis described above. The sRNA reads were then mapped to wheat aphid EVE transcripts using Bowtie software v1.2.3 with a perfect match [36]. Subsequent analyses were performed using Linux bash scripts.

## Results

### Diversity of RNA viruses identified in three wheat aphid species

**Fig. 1:**
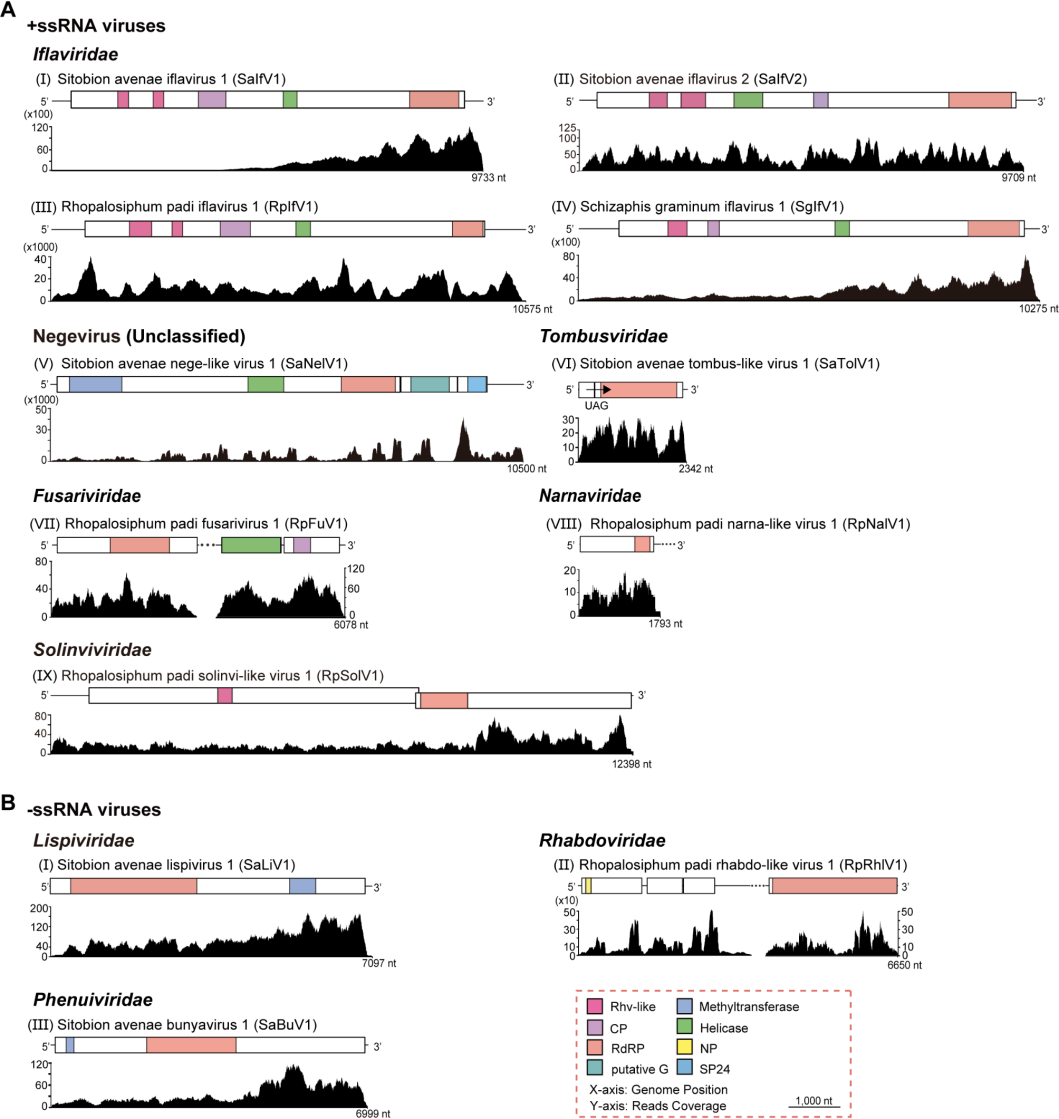
Genomic structures of novel RNA viruses identified in the three wheat aphid species. The viruses were classified into two groups: +ssRNA viruses (**A**) and -ssRNA viruses (**B**). Each virus was listed according to its taxonomic family. Conserved functional domains are color-coded, with the domain names indicated at the bottom of the figure. RdRP, RNA-dependent RNA polymerase; CP, coat protein; G, glycoprotein; NP, nucleoprotein. GenBank accession numbers are listed in Table 1

**Fig. 2:**
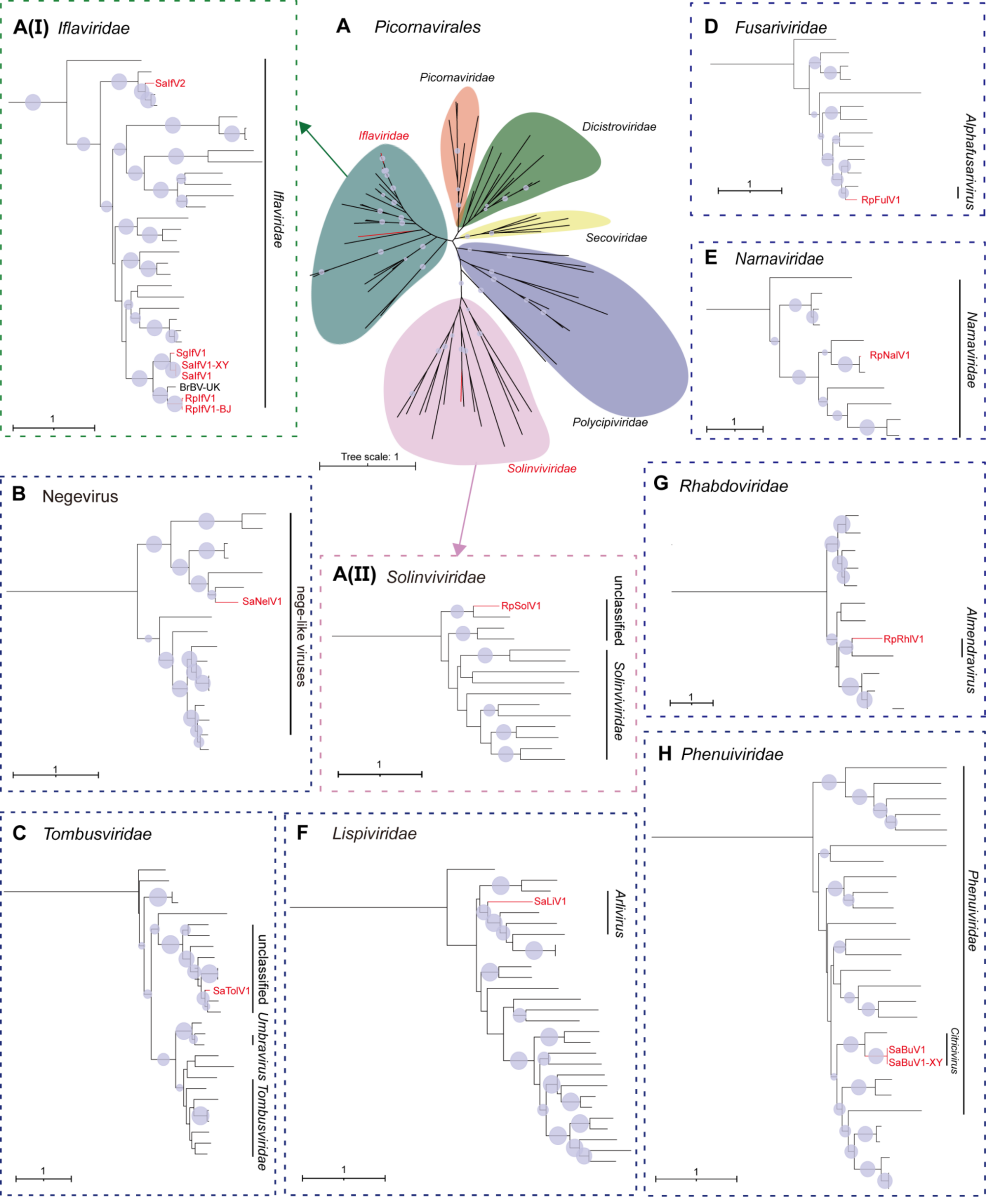
Phylogenetic analysis of the identified wheat aphid RNA viruses. The phylogenetic tree for *Iflaviridae* [**A**(I)], *Solinviviridae* [**A**(II)], Negevirus (**B**), *Tombusviridae* (**C**), *Fusariviridae* (**D**), *Narnaviridae* (**E**), *Lispiviridae* (**F**), *Rhabdoviridae* (**G**) and *Phenuiviridae* (**H**) are based on the maximum likelihood method and inferred from conserved viral RdRP domains. Novel RNA viruses are shown in red font. Nodes with bootstrap values > 50% are marked with blue circles. In panels A, taxonomic overview of the order *Picornavirales* is shown in the center, and close-up views of two clusters shown in the dashed boxes with arrows. The detailed virus names in each branch of the phylogenetic tree are shown in Fig. S1 and the GenBank accession numbers of these viruses are listed in Table S4

A total of 123 selected datasets of three wheat aphid species from the NCBI SRA repository were reassembled. Besides, field samples of *S. avenae* were collected and performed RNA-seq on the aphid samples. All of the wheat aphid datasets were subsequently used for the RNA virome analysis (Table S1). Among all the assembled libraries, 43 RNA viruses with complete RNA-dependent RNA polymerase (RdRP) domains were identified, including 10 novel ISVs, two novel mycoviruses, 27 known ISVs and four plant viruses that have been reported previously (Table 1 and Table S3). Based on the genome organization and phylogenetic analysis, 12 novel RNA viruses were classified into 10 different viral families. These included nine + ssRNA viruses (*Iflaviridae*, Negevirus, *Tombusviridae*, *Fusariviridae*, *Narnaviridae*, and *Solinviviridae*) and three -ssRNA viruses (*Lispiviridae*, *Rhabdoviridae* and *Phenuiviridae*). The genome organization and transcriptome raw read coverage of these ISVs are shown in Fig. 1. The classification of these viruses was determined by phylogenetic analyses based on predicted viral RdRP protein sequences (Fig. 2).

**Table 1:** Twelve novel RNA viruses identified in aphid species from public database and field-collected samples

Tentative virus names	NCBI Accession	Length (nt)	Coverage	E-value	Homologous virus	Protein identities	Virus family	Virus genus
Sitobion avenae iflavirus 1 (SaIfV1)	PQ181522	9733	1201.3	0.0	Brevicoryne brassicae virus	39.71%	*Iflaviridae*	*Iflavirus*
Sitobion avenae iflavirus 2 (SaIfV2)	BK068970	9709	42.3	0.0	Pityohyphantes rubrofasciatus iflavirus	65.05%	*Iflaviridae*	Unknow
Rhopalosiphum padi iflavirus 1 (RpIfV1)	BK068971	10,575	5761.3	0.0	Brevicoryne brassicae virus - UK	58.60%	*Iflaviridae*	*Iflavirus*
Schizaphis graminum iflavirus 1 (SgIfV1)	BK068973	10,275	726.3	0.0	Brevicoryne brassicae virus - UK	37.72%	*Iflaviridae*	*Iflavirus*
Sitobion avenae nege-like virus 1 (SaNelV1)	BK068977	10,500	9000.3	0.0	Wuhan insect virus 8	49.45%	Negevirus	Unknow
Sitobion avenae tombus-like virus 1 (SaTolV1)	BK068978	234	8.3	0.0	Verticillium dahliae RNA virus	72.27%	*Tombusviridae*	Unknow
Rhopalosiphum padi fusarivirus 1 (RpFuV1) *	BK068981	6078	25.8	0.0	Penicillium roqueforti ssRNA mycovirus 1	58.43%	*Fusariviridae*	*Alphafusarivirus*
Rhopalosiphum padi narna-like virus 1 (RpNalV1) *	BK068980	1793	4.6	0.0	Streptophyte associated narna-like virus 9	75.55%	*Narnaviridae*	Unknow
Rhopalosiphum padi Solinvi-like virus 1 (RpSolV1)	BK068974	12,398	16.9	0.0	Lasius neglectus picorna-like virus 3	40.20%	*Picornaviridae*	*Solinviviridae*
Sitobion avenae lispivirus 1 (SaLiV1)	BK068975	7097	31.6	0.0	Hemipteran arli-related virus OKIAV95	34.45%	*Lispiviridae*	Unknow
Rhopalosiphum padi rhabdo-like virus 1 (RpRhlV1)	BK068979	6650	62.0	0.0	Soybean thrips rhabdo-like virus 2	40.58%	*Rhabdoviridae*	*Almendravirus*
Sitobion avenae bunyavirus 1 (SaBuV1)	BK068982	6999	16.6	0.0	Aphis citricidus bunyavirus	44.52%	*Phenuiviridae*	*Citricivirus*

* Mycovirus identified in aphid samples

### +ssRNA viruses

+ssRNA viruses were the dominant type of wheat aphid RNA viruses due to the diversity and abundance. Nine novel + ssRNA viruses were identified in wheat aphids, which were classified into six families (Fig. 1A; Table 1). Five novel picornaviruses were found in the three wheat aphid species, belonging to the families *Iflaviridae* (Sitobion avenae iflavirus 1, SaIfV1; Sitobion avenae iflavirus 2, SaIfV2; Rhopalosiphum padi iflavirus 1, RpIfV1; and Schizaphis graminum iflavirus 1, SgIfV1) and *Solinviviridae* (Rhopalosiphum padi solinvi-like virus 1, RpSolV1). SaIfV1, RpIfV1, and SgIfV1 exhibited relatively high abundance, as indicated by coverage levels [Fig. 1A(I, III and IV)]. The RdRP sequences of these three iflaviruses had a protein identity of 37.72 – 58.60% (Table 1). They clustered closely with Brevicoryne brassicae virus-UK, forming a distinct clade within *Iflavirida*e (Fig. 2A). Another iflavirus SaIfV2 showed a 65.05% RdRP protein identity with Pityohyphantes rubrofasciatus iflavirus and clustered with other ISVs in in the family *Iflaviridae* [Fig. 1A(II) and 2A(I)]. A novel nege-like virus, Sitobion avenae nege-like virus 1 (SaNelV1), showing a 49.45% RdRP protein identity with Wuhan insect virus 8 was detected (Table 1) and clustered with other ISVs in an unclassified clade within the Negevirus [Fig. 1A(V) and 2B]. A novel Sitobion avenae tombus-like virus 1 (SaTolV1) had a 72.27% protein identity to the RdRP of Verticillium dahliae RNA virus and exhibited structural similarities to the family *Tombusviridae* [Fig. 1A(VI) and Table 1]. The genome of SaTolV1 had a UAG stop codon at the termination of ORF1 and ORF2 was translated through a stop codon readthrough mechanism alongside ORF1. Phylogenetic analysis showed that SaTolV1 clustered with other ISVs in the family *Tombusviridae* (Fig. 2C). A novel virus related to fungi, Rhopalosiphum padi fusarivirus 1 (RpFuV1), was identified belonging to the families of *Fusariviridae*. RpFuV1 showed a 58.43% RdRP protein identity with Penicillium roqueforti ssRNA mycovirus 1 and clustered with other mycoviruses in the family *Fusariviridae* [Fig. 1A(VII) and 2D]. Another novel virus, Rhopalosiphum padi narna-like virus 1 (RpNalV1), was detected with a 75.55% aa identity to the RdRP of Streptophyte-associated narna-like virus 9 and clustered closely with other mycoviruses in the family *Narnaviridae* [Fig. 1A(VIII) and 2E]. RpSolV1 exhibited a similar aa identity of 40.20% to the RdRP of Lasius neglectus picorna-like virus 3 and clearly clustered with other ISVs in the family *Solinviviridae* [Fig. 1A(IX) and 2A(II)].

To investigate the role of host siRNA-mediated immune regulation, we performed small RNA sequencing on field-collected of *S. avenae*. Extensive read coverage throughout the genome and the presence of small RNAs confirmed that SaIfV1 exhibited robust replication in *S. avenae* (Fig. S2). Moreover, virus-derived small interfering RNA (vsiRNA) of SaIfV1 were mainly distributed at a peak of 22 nt and showed a strong U bias at the 5′-terminal nucleotide (Fig. S2).

### -ssRNA viruses

Three novel negative-sense ssRNA viral genomes were identified within various wheat aphid datasets. A novel Sitobion avenae lispivirus 1 (SaLiV1), showing 34.45% RdRP protein identity to Hemipteran arli-related virus OKIAV95, clustered within a distinct branch closely related to the genus *Arlivirus* in the family *Lispiviridae* [Fig. 1B(I) and 2 F]. A novel Rhopalosiphum padi rhabdo-like virus 1(RpRhlV1), which exhibits 40.58% RdRP protein identity to Soybean thrips rhabdo-like virus 2, was classified within the genus *Almendravirus* (family *Rhabdoviridae*) [Fig. 1B(II) and 2G]. Another − ssRNA virus, Sitobion avenae bunyavirus 1 (SaBuV1), which is closely related to Aphis citricidus bunyavirus with RdRP aa sequence identity of 44.52%, grouped with other ISVs in the genus *Citricivirus* (family *Phenuiviridae*) [Fig. 1B(III) and 2 H].

### Abundance of RNA viruses in different geographic populations of three wheat aphid species

**Fig. 3:**
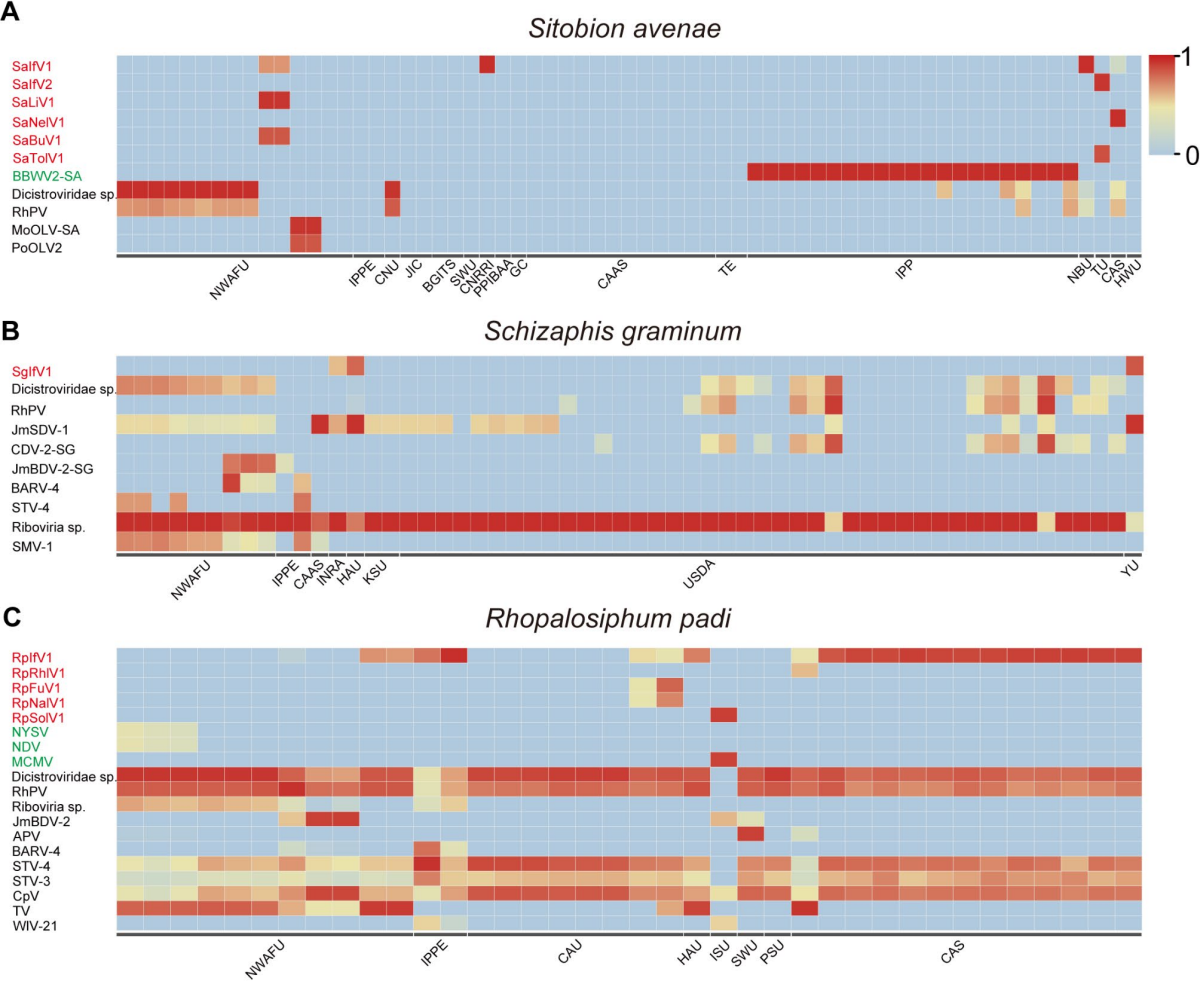
Analysis of RNA virome abundance in different datasets of three aphid species. Distribution of RNA viruses in *Sitobion avenae* (**A**), *Schizaphis graminum* (**B**), and *Rhopalosiphum padi* (**C**) across different databases. Blue indicates the absence of viral abundance, while other colors represent presence of viral abundance. The abundance ranges indicated by transcripts per million (TPM)

The abundance of viruses in three wheat aphid species (*S. avenae*, *S. graminum*, and *R. padi*) was then systematically investigated. In total, 11 viruses were identified in *S. avenae*, including six novel and five known viruses (Fig. 3A). *S. graminum* harbored ten viruses, comprising one novel and nine known viruses, while 19 viruses in *R. padi*, including five novel and 14 known viruses (Fig. 3B and C). The + ssRNA viruses were prevalent in the three aphid species with relatively high abundance (Fig. 3 and Table S3). 12 novel RNA viruses were identified in their respective species and several of these ISVs were only detected in limited geographic populations. For example, SaIfV2 and SaTolV1 were exclusively found in the *S. avenae* sample from Universidad de Talca (TU) with high abundance (Fig. 3A). RpFuV1 and RpNalV1 were present in *R. padi* sample from China Agricultural University (CAU) and RpSolV1 was detected only in *R. padi* sample submitted by the Iowa State University (ISU) (Fig. 3C). In contrast, several + ssRNA ISVs were present in the majority aphid populations, such as Dicistroviridae sp., Rhopalosiphum padi virus (RhPV) and Riboviria sp. For − ssRNA viruses, SaBuV1 was present solely in the aphid sample submitted by the Northwest A&F University (NWAFU) and RpRhlV1 was also identified in the *R. padi* sample from Chinese Academy of Sciences (CAS) with relatively high abundance (Fig. 3).

### Identification and phylogenetic analysis of EVEs in the genomes of three wheat aphids

**Fig. 4:**
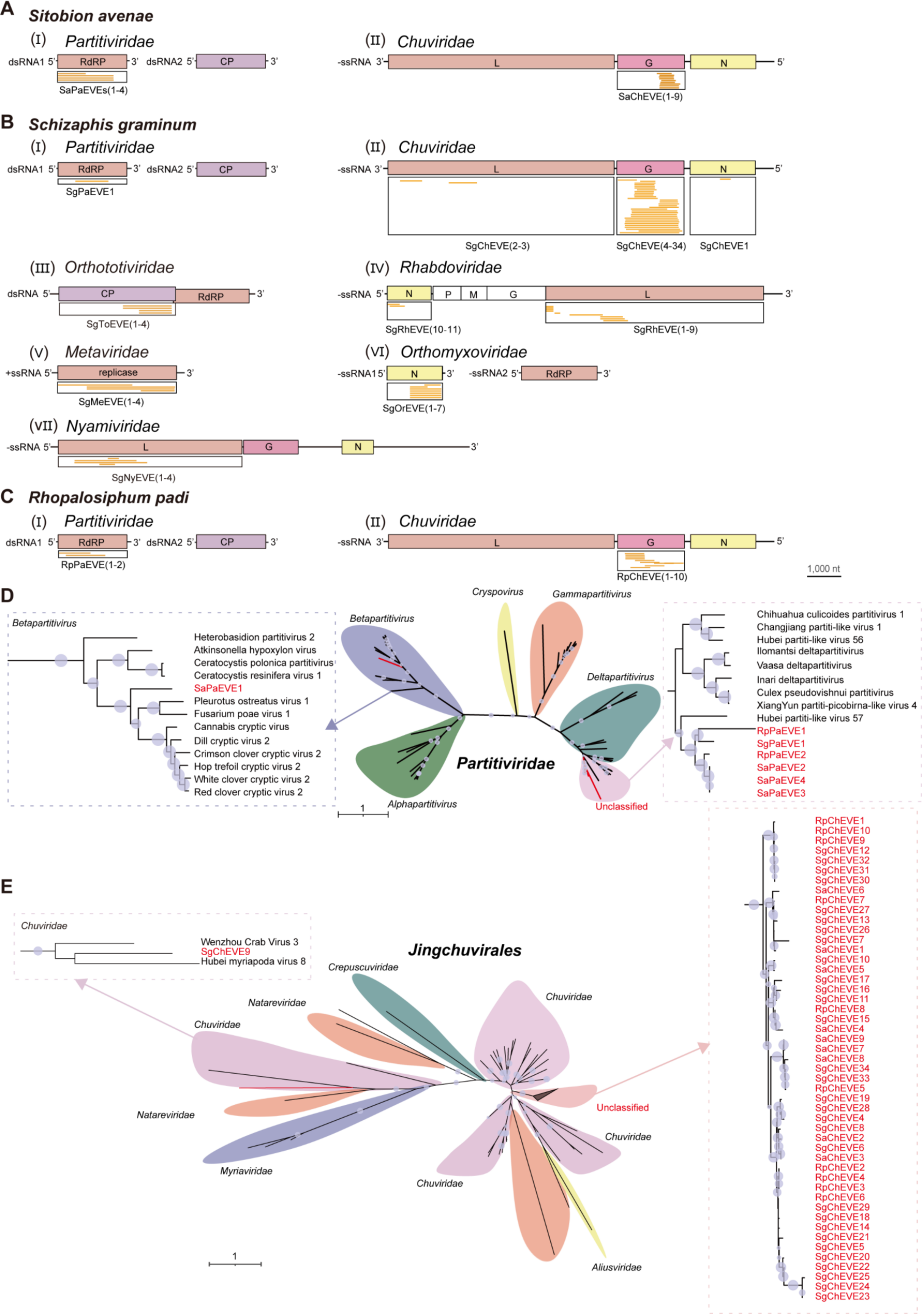
Identification of endogenous viral elements (EVEs) in the genomes of three wheat aphid species. (**A**–**C**) Alignment of EVEs from the genomes of *Sitobion avenae* (**A**), *Schizaphis graminum* (**B**), and *Rhopalosiphum padi* (**C**) with distinct viral genomes. The EVE names are shown below each type of viral genomes and the detailed information of these EVEs is listed in Table S5. RdRP, RNA-dependent RNA polymerase; L, large protein; CP, coat protein; G, glycoprotein; N, nucleoprotein. (**D** and **E**) Phylogenetic analysis of EVEs and related exogenous viruses. The maximum likelihood algorithm was used to construct phylogenetic trees for viral RdRP sequences in *Partitiviridae* (**D**) and **G** sequences in *Chuviridae* (**E**) and the corresponding EVE aa sequences. EVEs are shown in red font. Nodes with bootstrap values > 50% are marked with blue circles. Scale bars represent the percentage divergence. The viral sequences used for phylogenetic tree construction are listed in Table S4

To investigate EVEs in the genomes of three wheat aphids, the protein sequences of wheat aphid viruses identified in this study (Table 1 and Table S3) were used as queries to tBLASTn search against the genomes of the three wheat aphids. A total of 13, 65, and 12 EVEs were identified in the genomes of *S. avenae*, *S. graminum* and *R. padi*, respectively (Fig. 4 and Table S5). The 90 identified EVEs represent seven annotated virus families, including *Partitiviridae*, *Chuviridae*, *Nyamiviridae*, *Rhabdoviridae*, *Metaviridae*, *Orthototiviridae* and *Orthomyxoviridae*. EVEs derived from viruses in the *Chuviridae* and *Partitiviridae* families were found in all three aphid species (Fig. 4A and C). In addition, *Chuviridae*-derived EVEs (ChEVEs) were predominantly composed of glycoprotein (G), with smaller proportion originating from large protein (L) and nucleoprotein (N). EVEs derived from viruses in the *Partitiviridae* family (PaEVEs) were primarily composed of RdRP sequences. EVEs from viruses in the *Rhabdoviridae* and *Nyamiviridae* families were mainly originated from L protein coding sequences (Fig. 4B). Moreover, 20 EVE sequences of the three wheat aphid were selected and confirmed by PCR using specific primers, and the PCR products were further verified by Sanger sequencing (Fig. S3A).

Given that EVEs derived from viruses in the *Partitiviridae* and *Chuviridae* families were present in the genomes of the three aphid species, we constructed two phylogenetic trees to investigate the relationship between EVEs and their corresponding exogenous viruses. Seven PaEVEs and the RdRP proteins of the *Partitiviridae* family viruses were collected for phylogenetic analysis, and these PaEVEs were clearly divided into two clades (Fig. 4D). SaPaEVE1 was phylogenetically closely associated with mycoviruses (such as Pleurotus ostreatus virus 1 and Fusarium poae virus 1) and other six PaEVEs were closely related to ISVs (such as Hubei partiti-like virus 57) in the *Partitiviridae* family (Fig. 4D). Distance matrix analysis based on PaEVE amino acid sequences showed that SaPaEVE1 had relatively lower identities (18.8–24.3%) with other six PaEVEs, whereas the identities among six PaEVEs was relatively high (52.3–100.0%) (Table S6). Besides, the alignment of seven PaEVE sequences also showed that SaPaEVE1 was greatly varied with other six EVEs (Fig. S4A). For ChEVEs, the G proteins of viruses in different families of the order *Jingchuvirales* and 50 viral G protein-derived ChEVEs were collected for phylogenetic analysis. The results indicated that SgChEVE9 was clustered with arthropod viruses in the *Chuviridae*, while other ChEVEs were assigned to an unclassified clade (Fig. 4E). Meanwhile, SgChEVE9 had relatively low identities (12.3–19.2%) with other ChEVEs and was also differs greatly in aa sequence alignments (Table S7 and Fig. S4B). These results suggested that wheat aphid RNA viruses might have a long-term coexistence with wheat aphid hosts.

### Transcription expression analysis of EVEs in different geographic populations of three wheat aphid species

**Fig. 5:**
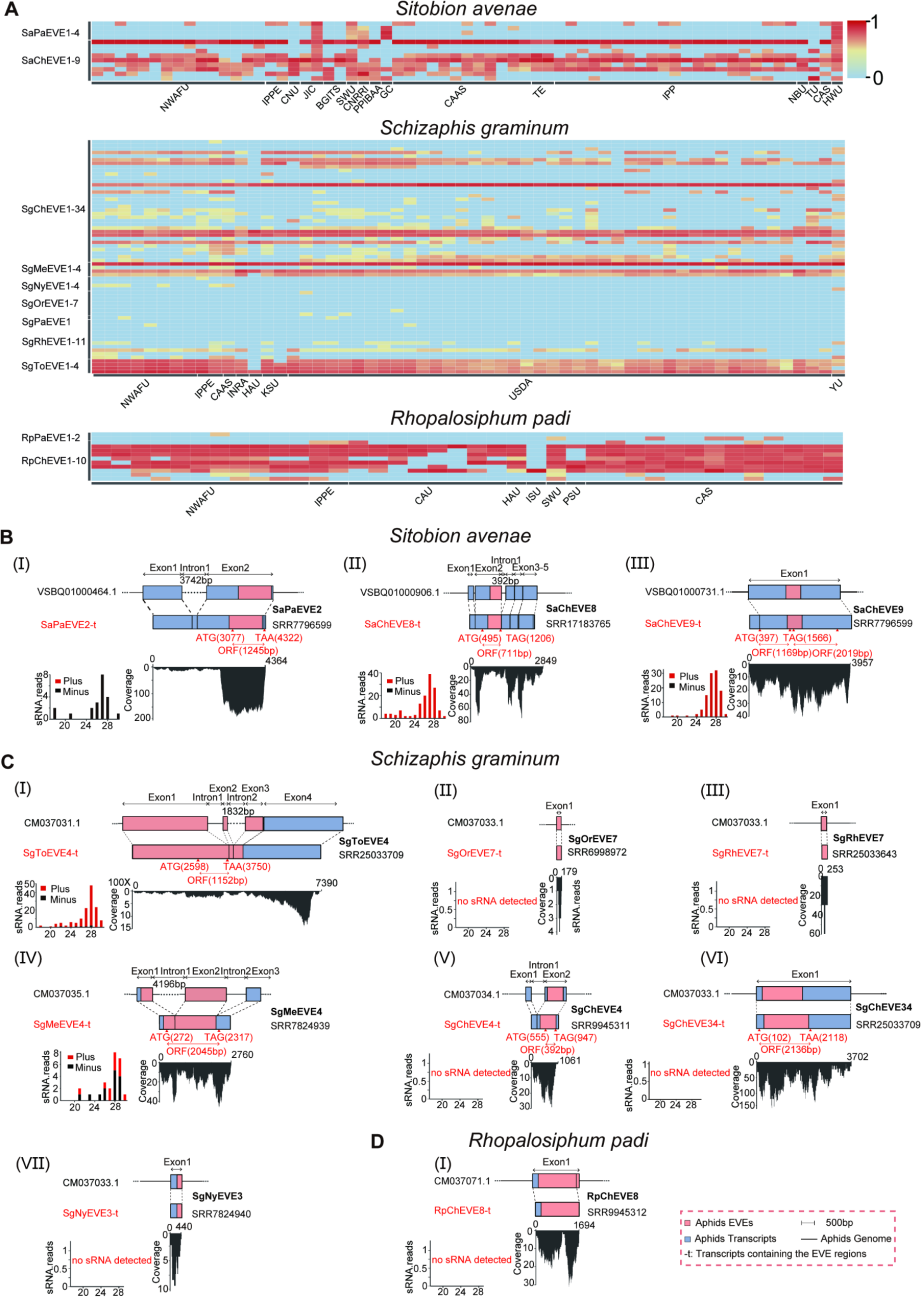
Transcription expression analyses of EVEs in different geographic populations of three wheat aphid species. (**A**) Heatmap displays the abundance of transcript reads derived from EVEs of *Sitobion avenae*, *Schizaphis graminum*, and *Rhopalosiphum padi* across wheat aphid datasets from various origins. Abbreviations for the datasets submitters and other related details are listed in Table S1. (**B**–**D**) Schematic diagrams represent the position and coverage of transcripts containing EVEs within the genome of *S. avenae* (**B**), *S. graminum* (**C**), and *R. padi* (**D**). The size distribution of small RNAs derived from each of the EVEs is displayed on the left panel. Predicted open reading frames (ORFs) are indicated with red double-headed arrows

To explore the potential transcription activity of the 90 identified EVEs, we screened a total of 161 publicly available wheat aphid transcriptomes (Table S1). The results showed that most EVEs were detected in at least one dataset and several EVEs, such as SaChEVE1, SgChEVE11, SgMeEVE2, SgToEVE(1–4), and RpChEVE(1–10), were widely distributed and expressed at relatively high levels in most aphid populations (Fig. 5A). Similarly, PaEVEs and ChEVEs in *S. avenae* were consistently expressed at high levels in both aphid populations submitted by the John Innes Centre (JIC) and University of Hawaii (HWU). Conversely, the expression of several EVEs was detected in limited aphid populations. For example, the expression of *Nyamiviridae*-derived EVEs (NyEVEs) was detected in *S. graminum* samples submitted by four laboratories and *Orthomyxoviridae*-derived EVEs (OrEVEs) were exclusively found in the sample from USDA-ARS Center for Grain and Animal Health Research (USDA) with low abundance (Fig. 5A).

Next, we examined the position of 11 EVEs that were derived from seven viral families in their respective aphid genomes. The results showed that EVEs were integrated within the intact ORF coding frame and were expressed in the assembled transcripts (Fig. 5B and D). Besides, five EVEs (SaPaEVE2, SaChEVE8, SgToEVE4 SgMeEVE4 and SgChEVE4) were embedded within five distinct transcripts that exhibit typical eukaryotic exon-intron structures (Fig. 5B and C). The expression of 11 EVEs at the transcript levels was further validated by RT-PCR followed with Sanger sequencing (Fig. S3B). Moreover, analysis of EVE-derived small RNA (sRNA) profiles showed that many aphid EVEs produced abundant sRNA reads with lengths ranging from 24 to 29 nt (such as SaChEVE8, SgToEVE4, and SgMeEVE4). Although relatively high transcript read counts, no sRNA was detected for four EVEs (SgOrEVE7, SgRhEVE7, SgNyEVE3 and RpChEVE8) (Fig. 5C and D). These results demonstrated that these EVEs might be exploited by aphid hosts and domesticated into a group of new genes with specific functions during long-term evolution.

## Discussion

The application of metavirome methods has significantly advanced the discovery of novel viruses in eukaryotes, particularly in arthropods such as mosquitoes, ticks, whiteflies, planthoppers and aphids [39–41]. Exploring RNA virus diversity has also led to the identification of EVEs in various eukaryotes, providing an extensive fossil record of the molecular arms race between RNA viruses and their hosts over millions of years [42]. In this study, we conducted a comprehensive and systematic analysis to identify and analyze RNA viruses and EVEs in three wheat aphid species based on the transcriptomic and genomic analyses. We identified 12 novel viruses, and 90 EVEs in wheat aphids, revealing a remarkable diversity of ISVs and a history of virus-host coevolution in wheat aphids.

The virome of wheat aphids identified in this study included ISVs and viruses associated with plant or symbiontic fungi hosts. For example, such as RpFuV1, RpNalV1 and SaTolV1 were clustered with other mycoviruses, suggesting they were derived from symbiotic fungi in aphids. SaBuV1 generally has three genome segments like other bunyaviruses [43]. We identified only one segment, possibly due to the low abundance of the remaining two segments. Besides, we compared known viruses that are present in three aphid species, revealing a relatively high viral sequence similarity (80.4–100%). This high similarity may contribute to the intraspecific diversity of viral sequences. In addition, four known plant viruses were identified in the three wheat aphid species: Broad bean wilt virus 2 (BBWV2), Maize chlorotic mottle virus (MCMV), Narcissus degeneration virus (NDV), and Narcissus yellow stripe virus (NYSV). These viruses are pathogenic to economically important crops or ornamental plants and are typically transmitted by aphids in a non-persistent manner [44–46]. Although wheat aphids have not been previously reported as vectors for these viruses, we identified nearly complete genomes for these four plant viruses (Fig. 3 and Table S3). The results showed that BBWV2 might be transmitted by *S. avenae*, and MCMV, NDV, NYSV might be transmitted by *R. padi*. However, the detection of these plant viruses could also result from contamination during aphid feeding.

EVEs, serving as genomic fossils, provide insights into ancient viral-host interactions, and the evolutionary timeline of viral infections [47, 48]. Analysis of EVE sequences reveals that various viruses can integrate into animal genomes, particularly in arthropods where EVEs are widespread [49–52]. For example, the investigation of endogenous nege-like virus elements has shed light on the history of negevirus infections embedded in aphid genomes. Analysis of negevirus-derived EVEs revealed the presence of one clade with orthologous sequences, suggesting an integration event is occurred approximately 63 to 81 million years ago (MYA). Another clade displayed non-orthologous integrations, which are considered to be independent endogenization events in the Cenozoic era [22]. In this study, 90 EVEs were identified in three wheat aphid species, most of which were derived from G protein of *Chuviridae* (Fig. 4). A similar trend of glycoprotein EVEs derived from *Jingchuvirales* has been reported in other species, including mosquitoes, silkworms, ants and beetles [53–55], indicating a bias in viral genes integrated as EVEs within their host genomes. Although EVEs derived from the G protein of *Chuviridae* and the RdRP of *Partitiviridae* were detected in all three wheat aphids, no orthologous EVEs were identified in these sequences, suggesting that these EVEs may have been independently integrated following the divergence of the three species. Besides, we have not found any aphid viruses from the families *Chuviridae* and *Partitiviridae*, making it challenging to assess the co-evolutionary relationships between aphid viruses and their corresponding EVEs. Further research can focus on the history of viral infection and the origins of ISVs in wheat aphid hosts.

More recently, several EVEs in arthropods have been reported to produce functional transcripts, including small RNAs, non-coding RNAs, and mRNAs [20, 20, 56, 57]. We also showed that most EVEs displayed transcriptional activity (Fig. 5A). Seven of the 11 selected EVEs were found to contain the complete ORFs (Fig. 5B and C). A similar phenomenon has been observed for totivirus-derived EVEs in rice planthoppers [24], indicating that these EVEs might be potentially tamed as authentic genes of the hosts. Intron gain and duplication are crucial steps for achieving functionality in horizontally transferred genes from bacteria to eukaryotes [58]. Considering that the seven EVEs were exclusively located within the last exon of predicted aphid ORFs, they have the potential to alter (modify, enhance, or attenuate) the current functions of aphid genes. It has been reported that several EVEs in arthropods exhibit transcriptional activity and produce piRNAs that can regulate homologous viral replication through a sequence-dependent RNA interfering pathway [59–61]. Nevertheless, experimental evidence of EVE-derived piRNAs mediating antiviral effects has so far been observed only in the ovaries of *Aedes aegypti* [62]. Furthermore, EVEs can exert antiviral effects through the expression of encoded proteins. For example, an EVE derived from the bornavirus nucleoprotein has been shown to inhibit Borna disease virus replication by encoding an antiviral protein. This EVE-encoded protein interferes with viral polymerase activity by binding to the viral ribonucleoprotein [63]. Although piRNA production was observed for the majority of transcribed EVEs (Fig. 5B and C), whether these EVEs are directly involved in aphid antiviral immunity against exogenous viruses remains to be further investigated.

In conclusion, we identified 12 novel RNA viruses and 90 EVEs in three wheat aphid species and analyzed the abundance of these viruses and EVEs in different populations of wheat aphids. Our findings provide valuable insights into the integration and domestication of viral elements within wheat aphid genomes, highlighting their potential functional roles.

## Electronic supplementary material

Below is the link to the electronic supplementary material.

**Suppelemtary Material 1: Fig S1.** Phylogenetic trees of the novel RNA viruses identified in three wheat aphids. Trees for *Solinviviridae *(A), *Iflaviridae *(B), *Phenuiviridae* (C), Negevirus (D), *Narnaviridae* (E), *Fusariviridae*(F), *Lispiviridae* (G), *Tombusviridae* (H) and  *Rhabdoviridae* (I) are based on the maximum likelihood method with conserved viral RdRP domains. Novel viruses are shown in red font

**Suppelemtary Material 2: Fig S2.** Profiles of virus derived small interfering RNAs (vsiRNAs) of SaIfV1. (**A**) the size distribution of SaIfV1-derived siRNAs. (**B**) 5’ terminal nucleotide preference of siRNAs derived from SaIfV1. (**C**) Distribution of SaIfV1-derived siRNA on the viral genome. Black represents siRNAs derived from the positive-sense strands, and red represents small RNAs derived from the negative-sense strands

**Suppelemtary Material 3: Fig S3.** Identification of EVEs in the genomes of three wheat aphid species by PCR and RT-PCR. (**A**) PCR confirmation of EVEs in the genome of three wheat aphid samples. (**B**) RT-PCR confirmation for the transcripts of EVEs in three wheat aphid samples

**Suppelemtary Material 4: Fig S4.** Predicted amino acid sequence alignment among wheat aphid EVEs derived viruses in the *Partitiviridae* (**A**) and *Chuviridae* (**B**) families

**Suppelemtary Material 5: Table S1.** Aphid datasets used in this study derived from public database and field investigation

**Suppelemtary Material 6: Table S2.** Primers used in this study

**Suppelemtary Material 7: Table S3.** Known ISVs and plant viruses identified in aphid species from the public database

**Suppelemtary Material 8: Table S4.** Detailed information on viruses used to construct phylogenetic trees

**Suppelemtary Material 9: Table S5.** Identification of endogenous viral elements (EVEs) in the genomes of the three wheat aphids

**Suppelemtary Material 10: Table S6.** Amino acid identities among *Partitiviridae*-derived EVEs

**Suppelemtary Material 11: Table S7.** Amino acid identities among *Chuviridae*-derived EVEs

## Data Availability

Data is provided within the manuscript or supplementary information files.

## References

[1] Hu XS, Liu XF, Thieme T, Zhang GS, Liu TX, Zhao HY. Testing the fecundity advantage hypothesis with *Sitobion avenae*, *Rhopalosiphum padi*, and *Schizaphis graminum* (Hemiptera: Aphididae) feeding on ten wheat accessions. Sci Rep. 2015;5:18549. 10.1038/srep18549 PMC4683512 26680508

[2] Ji X, Jiang YT, Guo TX, Zhang P, Li XA, Kong FB, Zhang BZ. Sublethal effects of imidacloprid on the fitness of two species of wheat aphids, *Schizaphis graminum* (R.) and *Rhopalosiphum padi* (L.). PloS one. 2023;18(11):e0294877. 10.1371/journal.pone.0294877 PMC10681248 38011174

[3] Zhang Z, Li Y, Li X, Zhu X, Zhang Y. Efficacy of Imidacloprid Seed Treatments against Four Wheat Aphids under Laboratory and Field Conditions. Plants (Basel). 2023;12(2). 10.3390/plants12020238 PMC9864830 36678951

[4] Jin Z, Wang X, Chang S, Zhou G. The complete nucleotide sequence and its organization of the genome of Barley yellow dwarf virus-GAV. Sci China C Life Sci. 2004;47(2):175–182. 10.1360/03yc0076 15379250

[5] Zhang W, Cheng Z, Xu L, Wu M, Waterhouse P, Zhou G, et al. The complete nucleotide sequence of the barley yellow dwarf GPV isolate from China shows that it is a new member of the genus Polerovirus. Arch Virol. 2009;154(7):1125–1128. 10.1007/s00705-009-0415-8 19551470

[6] Miller WA, Lozier Z. Yellow Dwarf Viruses of Cereals:Taxonomy and Molecular Mechanisms. Annu Rev Phytopathol. 2022;60:121–141. 10.1146/annurev-phyto-121421-125135 35436423

[7] Williamson C RE, Kasdorf GGF, Vonwechmar MB. Characterization of a new picorna-like virus isolated from aphids. Journal of General Virology. 1988;69,787–795.

[8] Liu S, Vijayendran D, Carrillo-Tripp J, Miller WA, Bonning BC. Analysis of new aphid lethal paralysis virus (ALPV) isolates suggests evolution of two ALPV species. J Gen Virol. 2014;95(Pt 12):2809–2819. 10.1099/vir.0.069765-0 25170050

[9] van Munster M DA, Verbeek M, van den Heuvel JFJM, Clérivet A, van der Wilk F. Sequence analysis and genomic organization of Aphid lethal paralysis virus: a new member of the family Dicistroviridae. J Gen Virol. 2002;83(12):3131–3138. 10.1099/0022-1317-83-12-3131 12466490

[10] Moon JS DL, McCoppin NK, D’Arcy CJ, Jin H. Nucleotide sequence analysis shows that Rhopalosiphum padi virus is a member of a novel group of insect-infecting RNA viruses. Virology. 1998:243(241):254–265. 10.1006/viro.1998.9043 9527915

[11] Ryabov EV. A novel virus isolated from the aphid Brevicoryne brassicae with similarity to Hymenoptera picorna-like viruses. J Gen Virol. 2007;88(9):2590–2595. 10.1099/vir.0.83050-0 17698671

[12] Ryabov EV, Keane G, Naish N, Evered C, Winstanley D. Densovirus induces winged morphs in asexual clones of the rosy apple aphid, *Dysaphis plantaginea*. Proc Natl Acad Sci U S A. 2009;106(21):8465–8470. 10.1073/pnas.0901389106 PMC2688996 19439653

[13] Vanden Heuvel J HH, Verbeek M, Dullemans AM, vanderWilk F. Characteristics of Acyrthosiphon pisum virus, a newly identified virus infecting the pea aphid. J Invertebr Pathol. 1997;70,169–176. 10.1006/jipa.1997.4691 9367722

[14] Nouri S, Matsumura EE, Kuo YW, Falk BW. Insect-specific viruses: from discovery to potential translational applications. Curr Opin Virol. 2018;33:33–41. 10.1016/j.coviro.2018.07.006 30048906

[15] Kondo H, Fujita M, Hisano H, Hyodo K, Andika IB, Suzuki N. Virome Analysis of Aphid Populations That Infest the Barley Field: The Discovery of Two Novel Groups of Nege/Kita-Like Viruses and Other Novel RNA Viruses. Front Microbiol. 2020;11:509. 10.3389/fmicb.2020.00509 PMC7154061 32318034

[16] An X, Zhang W, Ye C, Smagghe G, Wang JJ, Niu J. Discovery of a widespread presence bunyavirus that may have symbiont-like relationships with different species of aphids. Insect Sci. 2022;29(4):1120–1134. 10.1111/1744-7917.12989 34874617

[17] Guo Y, Zhao Y, Yang Y, Zhang Y, Li Y, Tian H, et al. Plants affect the horizontal transmission of a new densovirus infecting the green peach aphid Myzus persicae by modulating honeydew production. Insect Sci. 2024;31(1):236–254. 10.1111/1744-7917.13235 37370252

[18] An X, Gu Q, Wang J, Chang T, Zhang W, Wang JJ, et al. Insect-specific RNA virus affects the stylet penetration activity of brown citrus aphid (*Aphis citricidus*) to facilitate its transmission. Insect Sci. 2024;31(1):255–270. 10.1111/1744-7917.13242 37358052

[19] Palatini U, Contreras CA, Gasmi L, Bonizzoni M. Endogenous viral elements in mosquito genomes: current knowledge and outstanding questions. Curr Opin Insect Sci. 2022;49:22–30. 10.1016/j.cois.2021.10.007 34740858

[20] Fort P, Albertini A, Van-Hua A, Berthomieu A, Roche S, Delsuc F, et al. Fossil rhabdoviral sequences integrated into arthropod genomes: ontogeny, evolution, and potential functionality. Mol Biol Evol. 2012;29(1):381–390. 10.1093/molbev/msr226 21917725

[21] Liu S, Coates BS, Bonning BC. Endogenous viral elements integrated into the genome of the soybean aphid, *Aphis glycines*. Insect Biochem Mol Biol. 2020;123:103405. 10.1016/j.ibmb.2020.103405 32534986

[22] Lu G, Ye ZX, Qi YH, Lu JB, Mao QZ, Zhuo JC, et al. Endogenous nege-like viral elements in arthropod genomes reveal virus-host coevolution and ancient history of two plant virus families. J Virol. 2024;98(10):e0099724. 10.1128/jvi.00997-24 PMC11494950 39212930

[23] Yang Q, Zhang Y, Andika IB, Liao Z, Kondo H, Lu Y, et al. Horizontal Transfer of a Retrotransposon from the Rice Planthopper to the Genome of an Insect DNA Virus. J Virol. 2019;93(6). 10.1128/JVI.01516-18 PMC6401454 30626674

[24] Huang HJ, Li YY, Ye ZX, Li LL, Hu QL, He YJ, et al. Co-option of a non-retroviral endogenous viral element in planthoppers. Nat Commun. 2023;14(1):7264. 10.1038/s41467-023-43186-2 PMC10636211 37945658

[25] Grabherr MG, Haas BJ, Yassour M, Levin JZ, Thompson DA, Amit I, et al. Full-length transcriptome assembly from RNA-Seq data without a reference genome. Nat Biotechnol. 2011;29(7):644–652. 10.1038/nbt.1883 PMC3571712 21572440

[26] Antipov D, Raiko M, Lapidus A, Pevzner PA. Metaviral SPAdes: assembly of viruses from metagenomic data. Bioinformatics. 2020;36(14):4126–4129. 10.1093/bioinformatics/btaa490 32413137

[27] Buchfink B, Xie C, Huson DH. Fast and sensitive protein alignment using DIAMOND. Nat Methods. 2015;12(1):59–60. 10.1038/nmeth.3176 25402007

[28] Mitchell AL, Attwood TK, Babbitt PC, Blum M, Bork P, Bridge A, et al. InterPro in 2019: improving coverage, classification and access to protein sequence annotations. Nucleic Acids Res. 2019;47(D1):D351-D360. 10.1093/nar/gky1100 PMC6323941 30398656

[29] Katoh K, Standley DM. MAFFT multiple sequence alignment software version 7: improvements in performance and usability. Mol Biol Evol. 2013;30(4):772–780. 10.1093/molbev/mst010 PMC3603318 23329690

[30] Talavera G CJ. Improvement of phylogenies after removing divergent and ambiguously aligned blocks from protein sequence alignments. Syst Biol. 2007;56(54):564–577. 10.1080/10635150701472164 17654362

[31] Darriba D, Posada D, Kozlov AM, Stamatakis A, Morel B, Flouri T. ModelTest-NG: A New and Scalable Tool for the Selection of DNA and Protein Evolutionary Models. Mol Biol Evol. 2020;37(1):291–294. 10.1093/molbev/msz189 PMC6984357 31432070

[32] Kozlov AM, Darriba D, Flouri T, Morel B, Stamatakis A. RAxML-NG: a fast, scalable and user-friendly tool for maximum likelihood phylogenetic inference. Bioinformatics. 2019;35(21):4453–4455. 10.1093/bioinformatics/btz305 PMC6821337 31070718

[33] Langmead B, Salzberg SL. Fast gapped-read alignment with Bowtie 2. Nat Methods. 2012;9(4):357–359. 10.1038/nmeth.1923 PMC3322381 22388286

[34] Quan J, Wu Z, Ye Y, Peng L, Wu J, Ruan D, et al. Metagenomic Characterization of Intestinal Regions in Pigs With Contrasting Feed Efficiency. Front Microbiol. 2020;11:32. 10.3389/fmicb.2020.00032 PMC6989599 32038603

[35] Wagner GP, Kin K, Lynch VJ. Measurement of mRNA abundance using RNA-seq data: RPKM measure is inconsistent among samples. Theory Biosci. 2012;131(4):281–285. 10.1007/s12064-012-0162-3 22872506

[36] Langmead B, Trapnell C, Pop M, Salzberg SL. Ultrafast and memory-efficient alignment of short DNA sequences to the human genome. Genome Biol. 2009;10(3):R25. 10.1186/gb-2009-10-3-r25 PMC2690996 19261174

[37] Clewley JPaA, C. MEGALIGN. The multiple alignment module of LASERGENE. Methods Mol Biol. 1997;70:119–129. 9089607

[38] Hall TA. BioEdit: A User-Friendly Biological Sequence Alignment Editor and Analysis Program for Windows 95/98/NT. Nuclc Acids Symposium Series. 1999;41(41):95–98.

[39] Parry R, James ME, Asgari S. Uncovering the Worldwide Diversity and Evolution of the Virome of the Mosquitoes *Aedes aegypti* and *Aedes albopictus*. Microorganisms. 2021;9(8). 10.3390/microorganisms9081653 PMC8398489 34442732

[40] He T, Zhu C, Li Z, Ai L, Hu D, Wang C, et al. Virome analysis of ticks in Zhoushan Archipelago, China. J Vet Med Sci. 2022;84(6):847–854. 10.1292/jvms.22-0058 PMC9246684 35584918

[41] Mao QZ, Ye ZX, Yuan JN, Ning C, Chen MN, Xu ZT, et al. Diversity and transmissibility of RNA viruses in the small brown planthopper, *Laodelphax striatellus*. J Virol. 2024;98(12):e0019124. 10.1128/jvi.00191-24 PMC11650995 39589138

[42] Guzman-Solis AA, Navarro MA, Avila-Arcos MC, Blanco-Melo D. A Glimpse into the Past: What Ancient Viral Genomes Reveal About Human History. Annu Rev Virol. 2023;10(1):49–75. 10.1146/annurev-virology-111821-123859 37268008

[43] Maes P, Adkins S, Alkhovsky SV, Avsic-Zupanc T, Ballinger MJ, Bente DA, et al. Taxonomy of the order Bunyavirales: second update 2018. Arch Virol. 2019;164(3):927–941. 10.1007/s00705-018-04127-3 PMC6581445 30663021

[44] Ferrer RM, Ferriol I, Moreno P, Guerri J, Rubio L. Genetic variation and evolutionary analysis of broad bean wilt virus 2. Arch Virol. 2011;156(8):1445–1450. 10.1007/s00705-011-0990-3 21625974

[45] Redinbaugh MG, Stewart LR. Maize Lethal Necrosis: An Emerging, Synergistic Viral Disease. Annu Rev Virol. 2018;5(1):301–322. 10.1146/annurev-virology-092917-043413 30059641

[46] Ohshima K, Nomiyama R, Mitoma S, Honda Y, Yasaka R, Tomimura K. Evolutionary rates and genetic diversities of mixed potyviruses in Narcissus. Infect Genet Evol. 2016;45:213–223. 10.1016/j.meegid.2016.08.036 27590715

[47] Aiewsakun P, Katzourakis A. Endogenous viruses: Connecting recent and ancient viral evolution. Virology. 2015;479–480:26–37. 10.1016/j.virol.2015.02.011 25771486

[48] Feschotte C, Gilbert C. Endogenous viruses: insights into viral evolution and impact on host biology. Nat Rev Genet. 2012;13(4):283–296. 10.1038/nrg3199 22421730

[49] Blair CD, Olson KE, Bonizzoni M. The Widespread Occurrence and Potential Biological Roles of Endogenous Viral Elements in Insect Genomes. Curr Issues Mol Biol. 2020;34:13–30. 10.21775/cimb.034.013 31167954

[50] Katzourakis A, Gifford RJ. Endogenous viral elements in animal genomes. PLoS Genet. 2010;6(11):e1001191. 10.1371/journal.pgen.1001191 PMC2987831 21124940

[51] Cheng RL, Li XF, Zhang CX. Nudivirus Remnants in the Genomes of Arthropods. Genome Biol Evol. 2020;12(5):578–588. 10.1093/gbe/evaa074 PMC7250505 32282886

[52] Francois S, Filloux D, Roumagnac P, Bigot D, Gayral P, Martin DP, et al. Discovery of parvovirus-related sequences in an unexpected broad range of animals. Sci Rep. 2016;6:30880. 10.1038/srep30880 PMC5013282 27600734

[53] Li CX, Shi M, Tian JH, Lin XD, Kang YJ, Chen LJ, et al. Unprecedented genomic diversity of RNA viruses in arthropods reveals the ancestry of negative-sense RNA viruses. eLife. 2015;4. 10.7554/eLife.05378 PMC4384744 25633976

[54] Dezordi FZ, Vasconcelos C, Rezende AM, Wallau GL. In and Outs of Chuviridae Endogenous Viral Elements: Origin of a Potentially New Retrovirus and Signature of Ancient and Ongoing Arms Race in Mosquito Genomes. Front Genet. 2020;11:542437. 10.3389/fgene.2020.542437 PMC7642597 33193616

[55] Dezordi FZ, Coutinho GB, Dias YJM, Wallau GL. Ancient origin of Jingchuvirales derived glycoproteins integrated in arthropod genomes. Genet Mol Biol. 2023;46(1):e20220218. 10.1590/1678-4685-GMB-2022-0218 PMC10084718 37036390

[56] Ballinger MJ, Taylor DJ. Evolutionary persistence of insect bunyavirus infection despite host acquisition and expression of the viral nucleoprotein gene. Virus Evol. 2019;5(2):vez017. 10.1093/ve/vez017 PMC6620529 31308960

[57] Whitfield ZJ, Dolan PT, Kunitomi M, Tassetto M, Seetin MG, Oh S, et al. The Diversity, Structure, and Function of Heritable Adaptive Immunity Sequences in the *Aedes aegypti* Genome. Curr Biol. 2017;27(22):3511–3519 e3517. 10.1016/j.cub.2017.09.067 PMC5698160 29129531

[58] Olson KE, Bonizzoni M. Nonretroviral integrated RNA viruses in arthropod vectors: an occasional event or something more? Curr Opin Insect Sci. 2017;22:45–53. 10.1016/j.cois.2017.05.010 28805638

[59] Suzuki Y, Frangeul L, Dickson LB, Blanc H, Verdier Y, Vinh J, et al. Uncovering the Repertoire of Endogenous Flaviviral Elements in Aedes Mosquito Genomes. Journal of virology. 2017;91(15). 10.1128/JVI.00571-17 PMC5512259 28539440

[60] Russo AG, Kelly AG, Enosi Tuipulotu D, Tanaka MM, White PA. Novel insights into endogenous RNA viral elements in Ixodes scapularis and other arbovirus vector genomes. Virus Evol. 2019;5(1):vez010. 10.1093/ve/vez010 PMC6580184 31249694

[61] Ter Horst AM, Nigg JC, Dekker FM, Falk BW. Endogenous Viral Elements Are Widespread in Arthropod Genomes and Commonly Give Rise to PIWI-Interacting RNAs. J Virol. 2019;93(6). 10.1128/JVI.02124-18 PMC6401445 30567990

[62] Suzuki Y, Baidaliuk A, Miesen P, Frangeul L, Crist AB, Merkling SH, et al. Non-retroviral Endogenous Viral Element Limits Cognate Virus Replication in *Aedes aegypti* Ovaries. Curr Biol. 2020;30(18):3495–3506 e3496. 10.1016/j.cub.2020.06.057 PMC7522710 32679098

[63] Fujino K, Horie M, Honda T, Merriman DK, Tomonaga K. Inhibition of Borna disease virus replication by an endogenous bornavirus-like element in the ground squirrel genome. Proceedings of the National Academy of Sciences of the United States of America. 2014;111(36):13175–13180. 10.1073/pnas.1407046111 PMC4246967 25157155

