# Supplementary figures and images for: Diversity and evolution analysis of RNA viruses in three wheat aphid species
Source: BMC Genomics. 2025 Apr 7;26:353. doi: 10.1186/s12864-025-11512-1 (PMC11978097; doi:10.1186/s12864-025-11512-1)

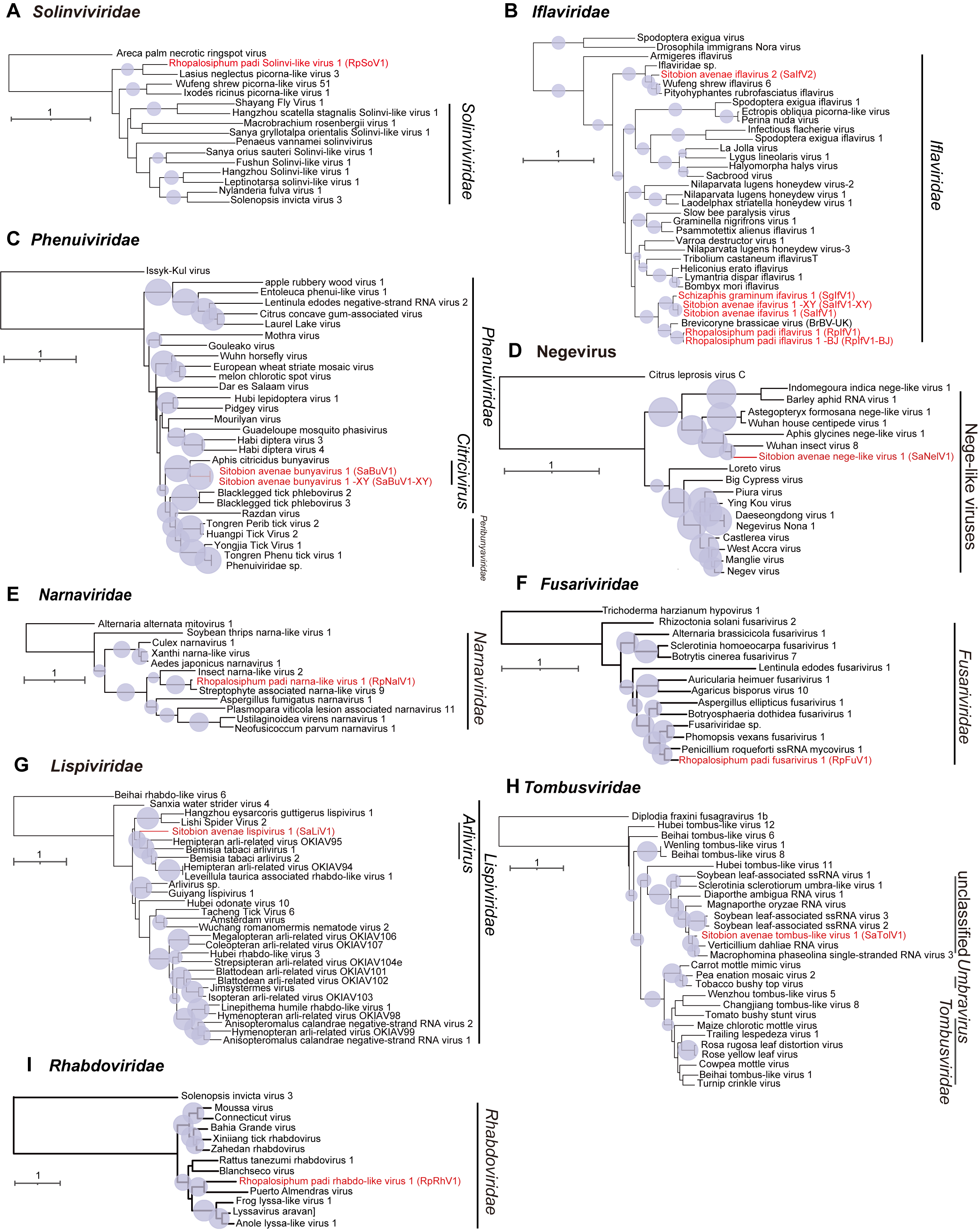

Supplement: Supplementary file 1 — Suppelemtary Material 1: Fig S1. Phylogenetic trees of the novel RNA viruses identified in three wheat aphids. Trees for Solinviviridae (A), Iflaviridae (B), Phenuiviridae (C), Negevirus (D), Narnaviridae (E), Fusariviridae(F), Lispiviridae (G), Tombusviridae (H) and Rhabdoviridae (I) are based on the maximum likelihood method with conserved viral RdRP domains. Novel viruses are shown in red font [file 12864_2025_11512_MOESM1_ESM.tif]

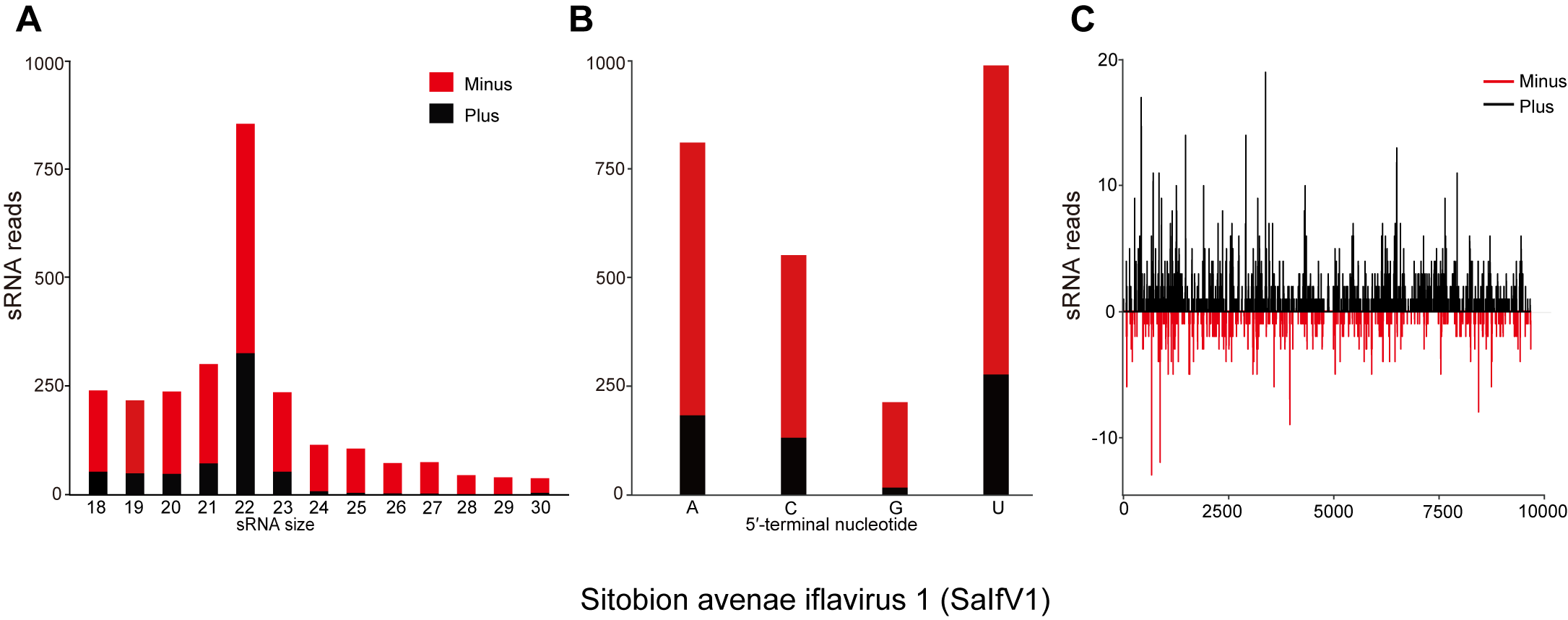

Supplement: Supplementary file 2 — Suppelemtary Material 2: Fig S2. Profiles of virus derived small interfering RNAs (vsiRNAs) of SaIfV1. (A) the size distribution of SaIfV1-derived siRNAs. (B) 5’ terminal nucleotide preference of siRNAs derived from SaIfV1. (C) Distribution of SaIfV1-derived siRNA on the viral genome. Black represents siRNAs derived from the positive-sense strands, and red represents small RNAs derived from the negative-sense strands [file 12864_2025_11512_MOESM2_ESM.tif]

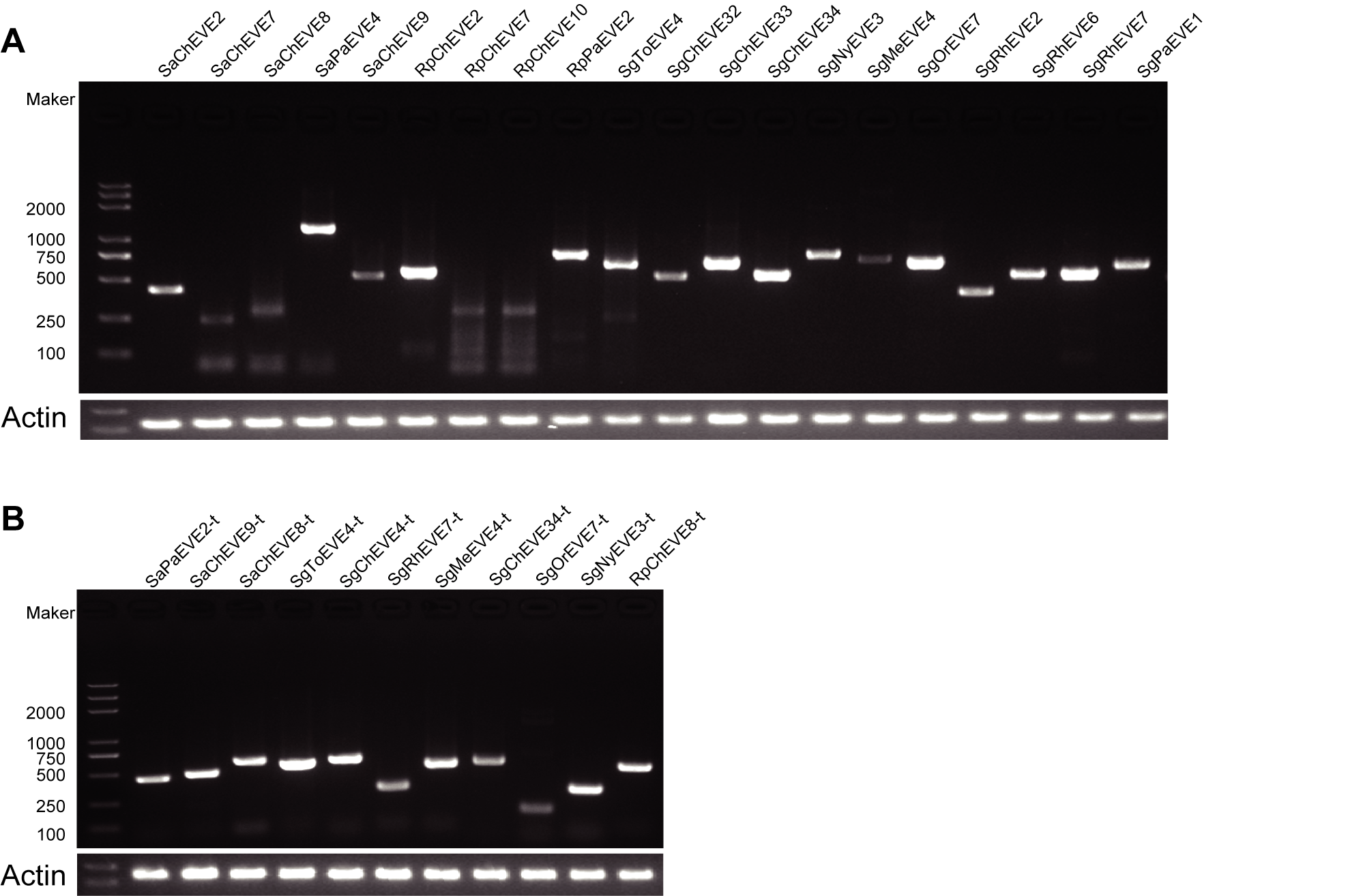

Supplement: Supplementary file 3 — Suppelemtary Material 3: Fig S3. Identification of EVEs in the genomes of three wheat aphid species by PCR and RT-PCR. (A) PCR confirmation of EVEs in the genome of three wheat aphid samples. (B) RT-PCR confirmation for the transcripts of EVEs in three wheat aphid samples [file 12864_2025_11512_MOESM3_ESM.tif]

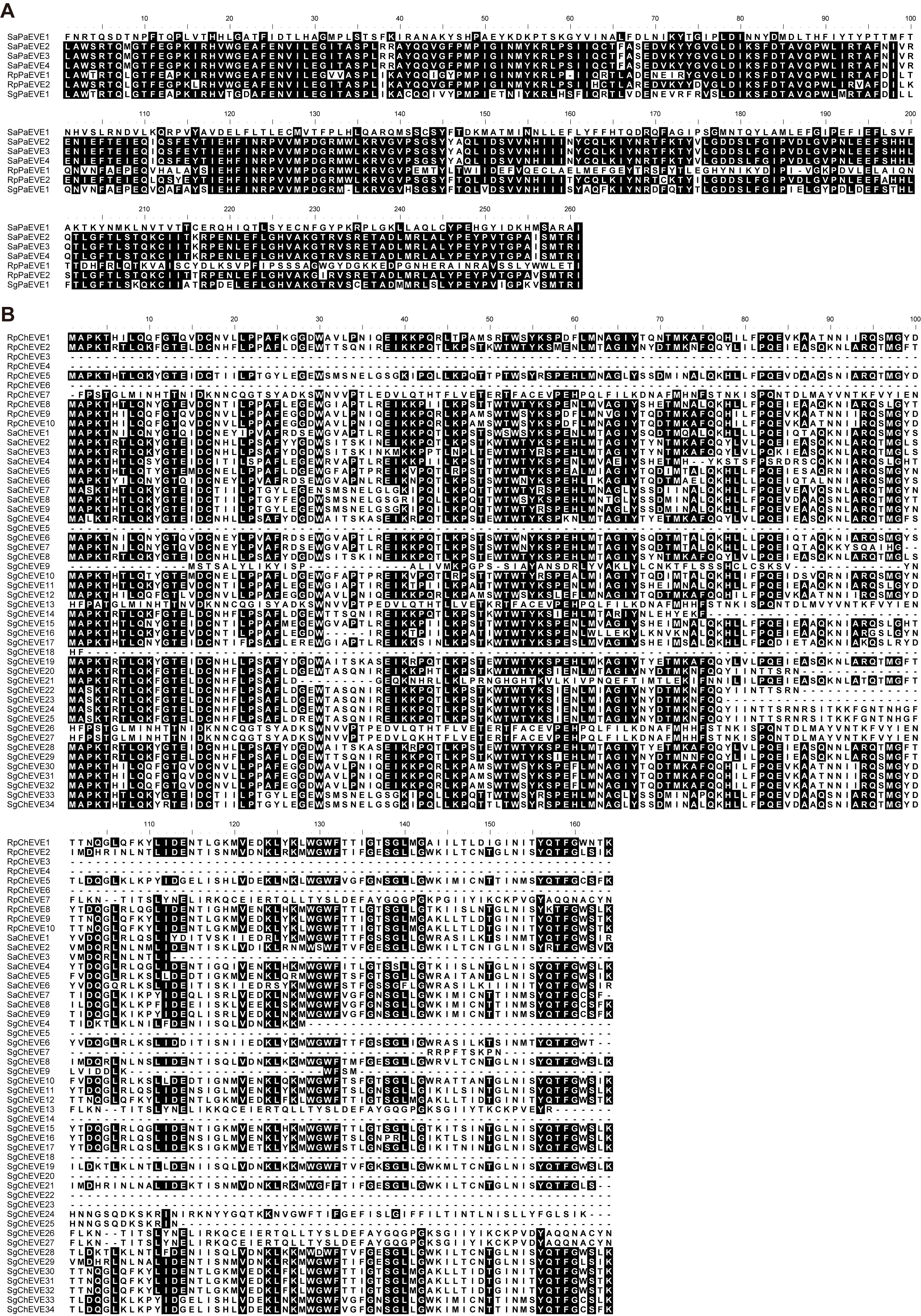

Supplement: Supplementary file 4 — Suppelemtary Material 4: Fig S4. Predicted amino acid sequence alignment among wheat aphid EVEs derived viruses in the Partitiviridae (A) and Chuviridae (B) families [file 12864_2025_11512_MOESM4_ESM.tif]
