# Supplementary material for: Diversity and evolution analysis of RNA viruses in three wheat aphid species
Source: BMC Genomics. 2025 Apr 7;26:353. doi: 10.1186/s12864-025-11512-1 (PMC11978097; doi:10.1186/s12864-025-11512-1)
Supplement: Supplementary file 5 — Suppelemtary Material 5: Table S1. Aphid datasets used in this study derived from public database and field investigation [file 12864_2025_11512_MOESM5_ESM.docx]

| **Table S1. Aphid datasets used in this study derived from public database and field investigation.** | | | | | |
| --- | --- | --- | --- | --- | --- |
| **Library** | **Accession  number** | **Institute** | **Description** | **Species** | **Size (GB)** |
| CNRRI | SRR11669716 | China National Rice Research Institute | Viral genomes in any invertebrate samples | *Sitobion avenae* | 13.1 |
| TE | ERR5987754 | Teagasc | RNA-seq of *Sitobion avenae* body parts  and developmental stages | *Sitobion avenae* | 10.6 |
| TE | ERR5987766 |  | RNA-seq of *Sitobion avenae* body parts  and developmental stages | *Sitobion avenae* | 10.6 |
| PPIBAA | SRR10295171 | Plant protection institute of Hebei academy of agr | Aphid and wheat transcriptome | *Sitobion avenae* | 7.6 |
| PPIBAA | SRR10295180 |  | Aphid and wheat transcriptome | *Sitobion avenae* | 7.6 |
| NWAFU | SRR10209592 | College of Plant Protection, Northwest A&F Univers | The transcriptome of *Sitobion avenae* biotypes  on different plants | *Sitobion avenae* | 8.6 |
| NWAFU | SRR10209596 |  | The transcriptome of *Sitobion avenae* biotypes on different plants | *Sitobion avenae* | 11.8 |
| NWAFU | SRR10209589 |  | The transcriptome of *Sitobion avenae* biotypes on different plants | *Sitobion avenae* | 8.2 |
| NWAFU | SRR10209595 |  | The transcriptome of *Sitobion avenae* biotypes on different plants | *Sitobion avenae* | 7.6 |
| NWAFU | SRR10209599 |  | The transcriptome of *Sitobion avenae* biotypes  on different plants | *Sitobion avenae* | 8.3 |
| NWAFU | SRR10872576 | Northwest A&F University | grain aphid transcriptome | *Sitobion avenae* | 8.0 |
| NWAFU | SRR10872581 |  | grain aphid transcriptome | *Sitobion avenae* | 7.8 |
| NWAFU | SRR7811388 |  | RNA-Seq of *Sitobion avenae* | *Sitobion avenae* | 8.8 |
| NWAFU | SRR7811385 |  | RNA-Seq of *Sitobion avenae* | *Sitobion avenae* | 7.8 |
| NWAFU | SRR7811386 |  | RNA-Seq of *Sitobion avenae* | *Sitobion avenae* | 6.9 |
| NWAFU | SRR7811387 |  | RNA-Seq of *Sitobion avenae* | *Sitobion avenae* | 7.1 |
| NWAFU | SRR7811389 |  | RNA-Seq of *Sitobion avenae* | *Sitobion avenae* | 7.4 |
| NWAFU | SRR7811390 |  | RNA-Seq of *Sitobion avenae* | *Sitobion avenae* | 6.6 |
| NWAFU | SRR7811391 |  | RNA-Seq of *Sitobion avenae* | *Sitobion avenae* | 6.3 |
| NWAFU | SRR7811392 |  | RNA-Seq of *Sitobion avenae* | *Sitobion avenae* | 6.1 |
| NWAFU | SRR7811393 |  | RNA-Seq of *Sitobion avenae* | *Sitobion avenae* | 6.3 |
| NWAFU | SRR7540990 |  | whole-transcriptome of Sitobion aveane  feeding on differeent host plants | *Sitobion avenae* | 6.8 |
| NWAFU | SRR7540989 |  | whole-transcriptome of Sitobion aveane  feeding on differeent host plants | *Sitobion avenae* | 6.6 |
| NBU | SRR30230442 | Ningbo University | RNA-Seq of *Sitobion avenae* | *Sitobion avenae* | 6.1 |
| CAAS | SRR14000461 | Chinese Academy of Agricultural Sciences | RNA-Seq of wingless *Sitobion avenae*: pseudo embryo | *Sitobion avenae* | 8.3 |
| CAAS | SRR14000449 |  | RNA-Seq of wingless *Sitobion avenae*: 1st instar nymph | *Sitobion avenae* | 8.7 |
| CAAS | SRR14000442 |  | RNA-Seq of wingless *Sitobion avenae*: 2nd instar nymph | *Sitobion avenae* | 8.0 |
| CAAS | SRR14000440 |  | RNA-Seq of wingless *Sitobion avenae*: 3nd instar nymph | *Sitobion avenae* | 7.4 |
| CAAS | SRR14000438 |  | RNA-Seq of wingless *Sitobion avenae*: 4th instar nymph | *Sitobion avenae* | 7.3 |
| CAAS | SRR14000458 |  | RNA-Seq of wingless *Sitobion avenae*: adult | *Sitobion avenae* | 7.3 |
| CAAS | SRR14000456 |  | RNA-Seq of wingled *Sitobion avenae*: pseudo embryo | *Sitobion avenae* | 8.0 |
| CAAS | SRR14000454 |  | RNA-Seq of wingled *Sitobion avenae*: 1st instar nymph | *Sitobion avenae* | 7.3 |
| CAAS | SRR14000453 |  | RNA-Seq of wingled *Sitobion avenae*: 2nd instar nymph | *Sitobion avenae* | 9.4 |
| CAAS | SRR14000451 |  | RNA-Seq of wingled *Sitobion avenae*: 3nd instar nymph | *Sitobion avenae* | 6.9 |
| CAAS | SRR14000447 |  | RNA-Seq of wingled *Sitobion avenae*: 4th instar nymph | *Sitobion avenae* | 7.2 |
| CAAS | SRR14000448 |  | RNA-Seq of wingled *Sitobion avenae*: 4th instar nymph | *Aphididae* | 6.8 |
| CAAS | SRR14000445 |  | RNA-Seq of wingled *Sitobion avenae*: adult | *Sitobion avenae* | 7.5 |
| CAAS | SRR11476040 |  | salivary gland transcriptome | *Sitobion avenae* | 8 |
| CAAS | SRR14000459 |  | RNA-Seq of wingless *Sitobion avenae*: adult | *Aphididae* | 6.6 |
| SWU | SRR17183765 | Southwest university | Trans rawdata from *Sitobion avenae* | *Sitobion avenae* | 8.2 |
| GC | SRR18731184 | Government of Canada | Targeted enrichment of *Sitobion avenae*  ultraconserved elements | *Sitobion avenae* | 2.3 |
| JIC | SRR21590299 | John Innes Centre | Strand-specific RNA-seq | *Sitobion avenae* | 14.8 |
| JIC | SRR21590300 |  | PCR-free wgs | *Sitobion avenae* | 30.1 |
| CNU | SRR25296178 | Chonnam National University | RNA-Seq of aphid: *Sitobion avenae* | *Sitobion avenae* | 7.7 |
| TU | SRR5373697 | Universidad de Talca | RNA-Seq of *Sitobion avenae*, less frequent clone | *Sitobion avenae* | 27.3 |
| TU | SRR5373698 |  | RNA-Seq of *Sitobion avenae*, frequent clone | *Sitobion avenae* | 27.3 |
| CAS | SRR5642366 | Institute of Zoology,  Chinese Academy of Science | *Sitobion avenae* | *Sitobion avenae* | 4.9 |
| BGITS | SRR648400 | BGITS-RNA | Chuli(upon feeding) | *Sitobion avenae* | 2.3 |
| BGITS | SRR648401 | BGITS-RNA | Duizhao(without feeding) | *Sitobion avenae* | 2.4 |
| HWU | SRR7796599 | University of Hawaii | HiSeq of endosymbiont Buchnera aphidicola from *Sitobion avenae* | *Sitobion avenae* | 3.7 |
| IPP | SRR8953738 | Institute of Plant Protection  and Agro-Products Sa | Transcriptome analysis and identification of  insecticidemetabolism related genes after  exposure to insecticide in *Sitobion avenae* | *Sitobion avenae* | 5.9 |
| IPP | SRR8953737 |  | Transcriptome analysis and identification of  insecticidemetabolism related genes after  exposure to insecticide in *Sitobion avenae* | *Sitobion avenae* | 6.0 |
| IPP | SRR8953740 |  | Transcriptome analysis and identification of  insecticidemetabolism related genes after  exposure to insecticide in *Sitobion avenae* | *Sitobion avenae* | 7.6 |
| IPP | SRR8953739 |  | Transcriptome analysis and identification of  insecticidemetabolism related genes after  exposure to insecticide in *Sitobion avenae* | *Sitobion avenae* | 7.0 |
| IPP | SRR8953742 |  | Transcriptome analysis and identification of  insecticidemetabolism related genes after  exposure to insecticide in *Sitobion avenae* | *Sitobion avenae* | 7.0 |
| IPP | SRR8953741 |  | Transcriptome analysis and identification of  insecticidemetabolism related genes after  exposure to insecticide in *Sitobion avenae* | *Sitobion avenae* | 7.0 |
| IPP | SRR8953744 |  | Transcriptome analysis and identification of  insecticidemetabolism related genes after  exposure to insecticide in *Sitobion avenae* | *Sitobion avenae* | 6.0 |
| IPP | SRR8953743 |  | Transcriptome analysis and identification of  insecticidemetabolism related genes after  exposure to insecticide in *Sitobion avenae* | *Sitobion avenae* | 7.8 |
| IPP | SRR8953755 |  | Transcriptome analysis and identification of  insecticidemetabolism related genes after  exposure to insecticide in *Sitobion avenae* | *Sitobion avenae* | 6.1 |
| IPP | SRR8953745 |  | Transcriptome analysis and identification of  insecticidemetabolism related genes after  exposure to insecticide in *Sitobion avenae* | *Sitobion avenae* | 7.9 |
| IPP | SRR8953752 |  | Transcriptome analysis and identification of  insecticidemetabolism related genes after  exposure to insecticide in *Sitobion avenae* | *Sitobion avenae* | 6.8 |
| IPP | SRR8953751 |  | Transcriptome analysis and identification of  insecticidemetabolism related genes after  exposure to insecticide in *Sitobion avenae* | *Sitobion avenae* | 7.9 |
| IPP | SRR8953750 |  | Transcriptome analysis and identification of  insecticidemetabolism related genes after  exposure to insecticide in *Sitobion avenae* | *Sitobion avenae* | 7.1 |
| IPP | SRR8953749 |  | Transcriptome analysis and identification of  insecticidemetabolism related genes after  exposure to insecticide in *Sitobion avenae* | *Sitobion avenae* | 6.8 |
| IPP | SRR8953754 |  | Transcriptome analysis and identification of  insecticidemetabolism related genes after  exposure to insecticide in *Sitobion avenae* | *Sitobion avenae* | 8.7 |
| IPP | SRR8953753 |  | Transcriptome analysis and identification of  insecticidemetabolism related genes after  exposure to insecticide in *Sitobion avenae* | *Sitobion avenae* | 7.1 |
| IPP | SRR8953736 |  | Transcriptome analysis and identification of  insecticidemetabolism related genes after  exposure to insecticide in *Sitobion avenae* | *Sitobion avenae* | 7.7 |
| IPP | SRR8953735 |  | Transcriptome analysis and identification of  insecticidemetabolism related genes after  exposure to insecticide in *Sitobion avenae* | *Sitobion avenae* | 8.0 |
| IPP | SRR8953748 |  | Transcriptome analysis and identification of  insecticidemetabolism related genes after  exposure to insecticide in *Sitobion avenae* | *Sitobion avenae* | 7.4 |
| IPP | SRR8953747 |  | Transcriptome analysis and identification of  insecticidemetabolism related genes after  exposure to insecticide in *Sitobion avenae* | *Sitobion avenae* | 7.3 |
| IPP | SRR8953746 |  | Transcriptome analysis and identification of  insecticidemetabolism related genes after  exposure to insecticide in *Sitobion avenae* | *Sitobion avenae* | 6.9 |
| IPPE | SRR9945314 | Institute of Plant Physiology  and Ecology, SIBS, C | Transcriptome of *Sitobion avenae* feed on  Triticum aestivum | *Sitobion avenae* | 7.0 |
| IPPE | SRR9945315 |  | Transcriptome of *Sitobion avenae* feed on  Trifolium repens | *Sitobion avenae* | 7.0 |
| CAU | SRR23300090 | China Agricultural University | RNAseq of *Rhopalosiphum padi* | *Rhopalosiphum padi* | 9.2 |
| CAU | SRR23300089 |  | RNAseq of *Rhopalosiphum padi* | *Rhopalosiphum padi* | 8.2 |
| CAU | SRR23300088 |  | RNAseq of *Rhopalosiphum padi* | *Rhopalosiphum padi* | 8.1 |
| CAU | SRR23300087 |  | RNAseq of *Rhopalosiphum padi* | *Rhopalosiphum padi* | 9.3 |
| CAU | SRR23300086 |  | RNAseq of *Rhopalosiphum padi* | *Rhopalosiphum padi* | 9.6 |
| CAU | SRR23300085 |  | RNAseq of *Rhopalosiphum padi* | *Rhopalosiphum padi* | 9.8 |
| CAU | SRR9722915 |  | transcriptome library of R. padi. | *Rhopalosiphum padi* | 7.0 |
| CAU | SRR9722916 |  | transcriptome library of R. padi. | *Rhopalosiphum padi* | 7.3 |
| CAU | SRR15365008 |  | RNA-Seq of *Rhopalosiphum padi*:Adult chest muscle | *Rhopalosiphum padi* | 6.2 |
| CAU | SRR9945312 | Institute of Plant Physiology  and Ecology, SIBS, C | Transcriptome of *Rhopalosiphum padi* feed on  Triticum aestivum | *Rhopalosiphum padi* | 8.4 |
| CAU | SRR9945313 |  | Transcriptome of *Rhopalosiphum padi* feed on  Trifolium repens | *Rhopalosiphum padi* | 8.5 |
| CAS | SRR18963159 | Institute of Zoology,  Chinese Academy of Sciences |  | *Rhopalosiphum padi* | 1.1 |
| CAS | SRR18963208 |  |  | *Rhopalosiphum padi* | 1.1 |
| CAS | SRR18963164 |  |  | *Rhopalosiphum padi* | 1.2 |
| CAS | SRR18963188 |  |  | *Rhopalosiphum padi* | 1.1 |
| CAS | SRR18963184 |  |  | *Rhopalosiphum padi* | 1.2 |
| CAS | SRR18963174 |  |  | *Rhopalosiphum padi* | 1.1 |
| CAS | SRR18963170 |  |  | *Rhopalosiphum padi* | 1.2 |
| CAS | SRR18963194 |  |  | *Rhopalosiphum padi* | 1.1 |
| CAS | SRR18963191 |  |  | *Rhopalosiphum padi* | 1.1 |
| CAS | SRR18963198 |  |  | *Rhopalosiphum padi* | 1.1 |
| CAS | SRR18963178 |  |  | *Rhopalosiphum padi* | 1.1 |
| CAS | SRR18963168 |  |  | *Rhopalosiphum padi* | 1.1 |
| CAS | SRR18963166 |  |  | *Rhopalosiphum padi* | 1.1 |
| CAS | SRR18963160 |  |  | *Rhopalosiphum padi* | 1.1 |
| CAS | SRR18963205 |  |  | *Rhopalosiphum padi* | 1.1 |
| CAS | SRR18963204 |  |  | *Rhopalosiphum padi* | 1.1 |
| CAS | SRR18963201 |  |  | *Rhopalosiphum padi* | 1.1 |
| CAS | SRR5642368 |  |  | *Rhopalosiphum padi* | 4.9 |
| CAS | SRR18963181 |  |  | *Rhopalosiphum padi* | 1.2 |
| CAS | SRR18963177 |  |  | *Rhopalosiphum padi* | 1.1 |
| ISU | SRR15538974 | Iowa State University | RNA-Seq of Zea mays field samples | *Rhopalosiphum padi* | 3.8 |
| NWAFU | SRR5133458 | Northwest A&F University | Rna-seq of *Rhopalosiphum padi*:adult | *Rhopalosiphum padi* | 9.9 |
| NWAFU | SRR5133471 |  | Rna-seq of *Rhopalosiphum padi*:adult | *Rhopalosiphum padi* | 9.9 |
| NWAFU | SRR7824936 |  | RNA-Seq of *Schizaphis graminum* and *Rhopalosiphum padi* | *Rhopalosiphum padi* | 6.8 |
| NWAFU | SRR7824943 |  | RNA-Seq of *Schizaphis graminum* and *Rhopalosiphum padi* | *Rhopalosiphum padi* | 6.5 |
| NWAFU | SRR7824944 |  | RNA-Seq of *Schizaphis graminum* and *Rhopalosiphum padi* | *Rhopalosiphum padi* | 7.4 |
| NWAFU | SRR7824941 |  | RNA-Seq of *Schizaphis graminum* and *Rhopalosiphum padi* | *Rhopalosiphum padi* | 8.5 |
| NWAFU | SRR7824929 |  | RNA-Seq of *Schizaphis graminum* and *Rhopalosiphum padi* | *Rhopalosiphum padi* | 6.7 |
| NWAFU | SRR7824942 |  | RNA-Seq of *Schizaphis graminum* and *Rhopalosiphum padi* | *Rhopalosiphum padi* | 7.5 |
| NWAFU | SRR7824945 |  | RNA-Seq of *Schizaphis graminum* and *Rhopalosiphum padi* | *Rhopalosiphum padi* | 7.2 |
| NWAFU | SRR7824946 |  | RNA-Seq of *Schizaphis graminum* and *Rhopalosiphum padi* | *Rhopalosiphum padi* | 7.0 |
| NWAFU | SRR7824938 |  | RNA-Seq of *Schizaphis graminum* and *Rhopalosiphum padi* | *Rhopalosiphum padi* | 6.5 |
| SWU | SRR17183779 | Southwest university | Trans rawdata from *Rhopalosiphum padi* | *Rhopalosiphum padi* | 8.1 |
| PSU | SRR3203855 | The Pennsylvania State University | Heat shock no infection | *Rhopalosiphum padi* | 4.7 |
| CAAS | SRR18308524 | Chinese Academy of Agricultural Sciences | RNA-Seq of Schizaphis gramimun: salivary gland | *Schizaphis graminum* | 9.3 |
| HAU | SRR15365009 | Henan Agricultural University | RNA-Seq of *Schizaphis graminum*:Adult chest muscle | *Schizaphis graminum* | 7.0 |
| INRA | SRR6118148 | INRA | Green bug salivary glands transcriptome | *Schizaphis graminum* | 0.2 |
| IPPE | SRR9945310 | Institute of Plant Physiology  and Ecology, SIBS, C | Transcriptome of *Schizaphis graminum* feed on  Trifolium aestivum | *Schizaphis graminum* | 8.2 |
| IPPE | SRR9945311 |  | Transcriptome of *Schizaphis graminum* feed on  Trifolium repens | *Schizaphis graminum* | 10.0 |
| KSU | SRR3038340 | Kansas State University |  | *Schizaphis graminum* | 0.4 |
| KSU | SRR3038325 |  |  | *Schizaphis graminum* | 0.4 |
| USDA | SRR6998972 | USDA-ARS Center for Grain  and Animal Health Resear | RNA-Seq of *Schizaphis graminum* | *Schizaphis graminum* | 6.1 |
| USDA | SRR6998978 |  | RNA-Seq of *Schizaphis graminum* | *Schizaphis graminum* | 7.6 |
| USDA | SRR6998965 |  | RNA-Seq of *Schizaphis graminum* | *Schizaphis graminum* | 6.0 |
| USDA | SRR6998954 |  | RNA-Seq of *Schizaphis graminum* | *Schizaphis graminum* | 6.9 |
| USDA | SRR6998964 |  | RNA-Seq of *Schizaphis graminum* | *Schizaphis graminum* | 6.0 |
| USDA | SRR6998958 |  | RNA-Seq of *Schizaphis graminum* | *Schizaphis graminum* | 6.0 |
| USDA | SRR6998967 |  | RNA-Seq of *Schizaphis graminum* | *Schizaphis graminum* | 6.2 |
| USDA | SRR6998975 |  | RNA-Seq of *Schizaphis graminum* | *Schizaphis graminum* | 5.9 |
| USDA | SRR6998984 |  | RNA-Seq of *Schizaphis graminum* | *Schizaphis graminum* | 7.8 |
| USDA | SRR25033709 | USDA-ARS-MWA | RNAseq of *Schizaphis graminum*: whole body with and without CYDV-RPV | *Schizaphis graminum* | 27.3 |
| USDA | SRR25033633 |  | RNAseq of *Schizaphis graminum*: whole body with and without CYDV-RPV | *Schizaphis graminum* | 20.1 |
| USDA | SRR25033642 |  | RNAseq of *Schizaphis graminum*: whole body with and without CYDV-RPV | *Schizaphis graminum* | 11 |
| USDA | SRR25033644 |  | RNAseq of *Schizaphis graminum*: whole body with and without CYDV-RPV | *Schizaphis graminum* | 20.1 |
| USDA | SRR25033675 |  | RNAseq of *Schizaphis graminum*: whole body with and without CYDV-RPV | *Schizaphis graminum* | 8.9 |
| USDA | SRR25033692 |  | RNAseq of *Schizaphis graminum*: whole body with and without CYDV-RPV | *Schizaphis graminum* | 29.3 |
| USDA | SRR25033686 |  | RNAseq of *Schizaphis graminum*: whole body with and without CYDV-RPV | *Schizaphis graminum* | 26.0 |
| USDA | SRR25033682 |  | RNAseq of *Schizaphis graminum*: whole body with and without CYDV-RPV | *Schizaphis graminum* | 25.8 |
| USDA | SRR25033679 |  | RNAseq of *Schizaphis graminum*: whole body with and without CYDV-RPV | *Schizaphis graminum* | 22.6 |
| USDA | SRR25033678 |  | RNAseq of *Schizaphis graminum*: whole body with and without CYDV-RPV | *Schizaphis graminum* | 11.8 |
| USDA | SRR25033673 |  | RNAseq of *Schizaphis graminum*: whole body with and without CYDV-RPV | *Schizaphis graminum* | 8.9 |
| USDA | SRR25033671 |  | RNAseq of *Schizaphis graminum*: whole body with and without CYDV-RPV | *Schizaphis graminum* | 28.6 |
| USDA | SRR25033667 |  | RNAseq of *Schizaphis graminum*: whole body with and without CYDV-RPV | *Schizaphis graminum* | 61.4 |
| USDA | SRR25033657 |  | RNAseq of *Schizaphis graminum*: whole body with and without CYDV-RPV | *Schizaphis graminum* | 27.7 |
| USDA | SRR25033650 |  | RNAseq of *Schizaphis graminum*: whole body with and without CYDV-RPV | *Schizaphis graminum* | 21.9 |
| USDA | SRR25033647 |  | RNAseq of *Schizaphis graminum*: whole body with and without CYDV-RPV | *Schizaphis graminum* | 19.6 |
| USDA | SRR25033643 |  | RNAseq of *Schizaphis graminum*: whole body with and without CYDV-RPV | *Schizaphis graminum* | 29.8 |
| USDA | SRR25033641 |  | RNAseq of *Schizaphis graminum*: whole body with and without CYDV-RPV | *Schizaphis graminum* | 11.2 |
| USDA | SRR25033638 |  | RNAseq of *Schizaphis graminum*: whole body with and without CYDV-RPV | *Schizaphis graminum* | 10.2 |
| USDA | SRR25033632 |  | RNAseq of *Schizaphis graminum*: whole body with and without CYDV-RPV | *Schizaphis graminum* | 24.2 |
| USDA | SRR25033628 |  | RNAseq of *Schizaphis graminum*: whole body with and without CYDV-RPV | *Schizaphis graminum* | 26.8 |
| USDA | SRR25033623 |  | RNAseq of *Schizaphis graminum*: whole body with and without CYDV-RPV | *Schizaphis graminum* | 23.7 |
| USDA | SRR25033618 |  | RNAseq of *Schizaphis graminum*: whole body with and without CYDV-RPV | *Schizaphis graminum* | 23.6 |
| USDA | SRR25033616 |  | RNAseq of *Schizaphis graminum*: whole body with and without CYDV-RPV | *Schizaphis graminum* | 8.9 |
| USDA | SRR25033702 |  | RNAseq of *Schizaphis graminum*: whole body with and without CYDV-RPV | *Schizaphis graminum* | 8.3 |
| USDA | SRR25033699 |  | RNAseq of *Schizaphis graminum*: whole body with and without CYDV-RPV | *Schizaphis graminum* | 28.5 |
| USDA | SRR25033696 |  | RNAseq of *Schizaphis graminum*: whole body with and without CYDV-RPV | *Schizaphis graminum* | 24.7 |
| USDA | SRR25033693 |  | RNAseq of *Schizaphis graminum*: whole body with and without CYDV-RPV | *Schizaphis graminum* | 21.8 |
| USDA | SRR25033690 |  | RNAseq of *Schizaphis graminum*: whole body with and without CYDV-RPV | *Schizaphis graminum* | 25.7 |
| USDA | SRR25033688 |  | RNAseq of *Schizaphis graminum*: whole body with and without CYDV-RPV | *Schizaphis graminum* | 24.9 |
| USDA | SRR25033664 |  | RNAseq of *Schizaphis graminum*: whole body with and without CYDV-RPV | *Schizaphis graminum* | 27.3 |
| USDA | SRR25033663 |  | RNAseq of *Schizaphis graminum*: whole body with and without CYDV-RPV | *Schizaphis graminum* | 9.7 |
| USDA | SRR25033658 |  | RNAseq of *Schizaphis graminum*: whole body with and without CYDV-RPV | *Schizaphis graminum* | 8.3 |
| USDA | SRR25033636 |  | RNAseq of *Schizaphis graminum*: whole body with and without CYDV-RPV | *Schizaphis graminum* | 27.6 |
| USDA | SRR25033626 |  | RNAseq of *Schizaphis graminum*: whole body with and without CYDV-RPV | *Schizaphis graminum* | 27.4 |
| YU | SRR935073 | Yale University | Buchnera Sg small RNA | *Schizaphis graminum* | 1.2 |
| NWAFU | SRR7824939 | Northwest A&F University | RNA-Seq of *Schizaphis graminum* and *Rhopalosiphum padi* | *Schizaphis graminum* | 7.3 |
| NWAFU | SRR7824940 |  | RNA-Seq of *Schizaphis graminum* and *Rhopalosiphum padi* | *Schizaphis graminum* | 7.2 |
| NWAFU | SRR7824930 |  | RNA-Seq of *Schizaphis graminum* and *Rhopalosiphum padi* | *Schizaphis graminum* | 7.0 |
| NWAFU | SRR7824932 |  | RNA-Seq of *Schizaphis graminum* and *Rhopalosiphum padi* | *Schizaphis graminum* | 7.1 |
| NWAFU | SRR7824934 |  | RNA-Seq of *Schizaphis graminum* and *Rhopalosiphum padi* | *Schizaphis graminum* | 8.2 |
| NWAFU | SRR7824935 |  | RNA-Seq of *Schizaphis graminum* and *Rhopalosiphum padi* | *Schizaphis graminum* | 8.1 |
| NWAFU | SRR7824931 |  | RNA-Seq of *Schizaphis graminum* and *Rhopalosiphum padi* | *Schizaphis graminum* | 7.6 |
| NWAFU | SRR7824933 |  | RNA-Seq of *Schizaphis graminum* and *Rhopalosiphum padi* | *Schizaphis graminum* | 7.2 |
| NWAFU | SRR7824937 |  | RNA-Seq of *Schizaphis graminum* and *Rhopalosiphum padi* | *Schizaphis graminum* | 7.1 |
| **miRNA datasets of three wheat aphid species retrieved from the NCBI SRA.** | | | | | |
| SWU | SRR17183773 | Southwest university | MiRNA rawdata from *Sitobion avenae* | *Sitobion avenae* | 2.2 |
| NBU | SRR30230441 | Ningbo University | MiRNA rawdata from *Sitobion avenae* | *Sitobion avenae* | 0.8 |
| UNL | SRR2014709 | University of Nebraska-Lincoln | miRNAs from *Schizaphis graminum* | *Schizaphis graminum* | 1.1 |
| SWU | SRR17183777 | Southwest university | MiRNA rawdata from *Rhopalosiphum padi* | *Rhopalosiphum padi* | 0.5 |
