# Supplementary material for: Diversity and evolution analysis of RNA viruses in three wheat aphid species
Source: BMC Genomics. 2025 Apr 7;26:353. doi: 10.1186/s12864-025-11512-1 (PMC11978097; doi:10.1186/s12864-025-11512-1)
Supplement: Supplementary file 6 — Suppelemtary Material 6: Table S2. Primers used in this study [file 12864_2025_11512_MOESM6_ESM.docx]

| **Table S2.** **Primers used in this study^1^.** | | | | |  |
| --- | --- | --- | --- | --- | --- |
| **Name** | **Forward primer (5’-3’)** | | **Reverse primer (5’-3’)** | |  |
| **SaIfV1 Primers used for RT-PCR in this study.** | | | | |  |
| SaIfV1 5′-RACE GSP | AATAAGTCTTGATCTTCCTGACTCA | | ACCGCAAATGGATCAAGGATCTCATGGTGTC | |  |
| SaIfV1 3′-RACE GSP | CCCCCACCCCACACGTAATGGTGTTTATC | | CTAATACGACTCACTATAGGGC | |  |
| SaIfV1 segment1 | GCCCGTGACCCAACCTATG | | CATTGAACTTTAACGAAGGC | |  |
| SaIfV1 segment2 | GCGAGCACCTTCACCCCAC | | CCCACCATCGTCAGCACA | |  |
| SaIfV1 segment3 | GGGGCTTCACCTACAACAG | | GGGTGCTCCATCTAACCGC | |  |
| SaIfV1 segment4 | GCGTGCATTAGGGGCAGCTGC | | AGTCCAGCTTTATTAGGAGG | |  |
| SaIfV1 segment5 | GCAGTCGTCCTATGTATCCTG | | GGGACAACAGAATTAACACTT | |  |
| SaIfV1 segment6 | GATCGAATAGCTAGAACATGG | | CGGACTATACCAAGGAACTTC | |  |
| SaIfV1 segment7 | GCAGAGATGGTGTTATACCT | | TGGCATCTCCACATAACGA | |  |
| SaIfV1 segment8 | GCGAATGAAACGGCGACAG | | CTTCCTGAGATTCCCCTTCC | |  |
| SaIfV1 segment9 | GCTTATTCTACTCATGCTCG | | CCTACTCCAGGTGGACCATG | |  |
| SaIfV1 segment10 | AACTATGGCGAGATGCAG | | CCCATGCCATATATCCCGG | |  |
| SaIfV1 segment11 | ATACGATTTCACCTGTAAC | | CATCAGATTTAATCATAGGCG | |  |
| SaIfV1 segment12 | CTCGAGAAGAATGTTGTACC | | CTAATAAGCTGCCCTTTGGCC | |  |
| SaIfV1 segment13 | GCTCGTAATATAGTTGTTAGTGC | | GGTATTCTACAATTGGGCCATCC | |  |
| SaIfV1 segment14 | CACAAATCTATCTCCGGATTG | | CGCGCTGCACCATATGCTGC | |  |
| SaIfV1 segment15 | CCCCTTTCTCGTATCCGCCCA | | GCGTAAGTGTAGGATCAACAC | |  |
| SaIfV1 segment16 | GTGGGATGCCCTCTGGTAATC | | GCACTATCTAATACAATACAGGC | |  |
| SaIfV1 segment17 | GTCCTGATATGAAAGGTGCTACG | | AGTCATATTAGCACTATCT | |  |
| **EVE Primers used in this study.** | | | | |  |
| SaChEVE2 | CATATTTCATGGCACCAAAAACG | | CCCAATAGACCAGAGACAAATTCT | |  |
| SaChEVE7 | CCAACAGGATATCTTATGGAAGG | | GCCCATTGTTTGTCTAGCTAAG | |  |
| SaChEVE8 | CGAATCCTACAAACCATCCCCAC | | GGTTCAGGTAGAATACCACAAC | |  |
| SaChEVE9 | CATGGCACCTAAAACACACACA | | GCTAGTACTTTGAAACTGCAGCC | |  |
| SaPaEVE4 | CGATACGTGTCATTGAGATTGC | | CCGAAACTCCGAAATGGACCG | |  |
| RpChEVE2 | GTATGCGAATTCGGATAAACTGTAT | | GTTAACGAGACTATATTATCATCCG | |  |
| RpChEVE7 | CTGAAATCATTAGTGTTGCTGG | | GACAAAAACGGAAACTGCCAAGG | |  |
| RpChEVE10 | CGTTAACTTTTTTGCATTTTATC | | GAGCAGTTATGGTTTTTCGTG | |  |
| RpPaEVE2 | CGGAAGACCAAGCGATCC | | GATGTAAGTCTTGCAGGTTCG | |  |
| SgChEVE32 | GACGCGCAAGTGGTGGATGG | | GGGATTCCTGTGCAAGCGAAC | |  |
| SgChEVE33 | CATGCTTGAAAATATACCCGC | | CGTACCAGAATTCGCATACTC | |  |
| SgChEVE34 | CCGAATCCTACAAACCAACC | | GCTTATCAGAATTCGCATACTC | |  |
| SgMeEVE4 | CGTCGGCAGGGGAGTTCC | | CGGAATACAGGCGGCGATA | |  |
| SgNyEVE3 | CAGCGCGTTCGTTATCACCCA | | CTTGCAAACTCAGGCCTCGTC | |  |
| SgOrEVE7 | CAATGTCCTTCTTCGCTGCAC | | AGCAAAAGGAGACGCTGAATCG | |  |
| SgRhEVE2 | GTCTCCTTGTGCAAGTTCTCGG | | GGATTGACAACCGTGGTGAAG | |  |
| SgRhEVE6 | GTATAGGATTCCGACTAAAGTCC | | CCGAGAATATGTCGTCTTATCAG | |  |
| SgRhEVE7 | GGATTCCGACTAAAGTCCACCCC | | CTGCAAGTTGACCTGACCACCC | |  |
| SgRhEVE9 | GGCCCGCGGATTTATTGTGGG | | GTATAGGAGATTTTTGCGGAC | |  |
| SgToEVE4 | GGCTTCACGACAGAAGTGCACC | | GGGCCGACAAACCTGTGGG | |  |
| SgPaEVE1 | CGGACAGACAGTCTGCCCTTTC | | GGGTCGGGTTGCGATAATGCAC | |  |
| SaPaEVE2-t | TGCCTCCGAAGACGTCAAAT | | AGGGCGCTTGGTGATGATAC | | |
| SaChEVE8-t | GGCACCCAAAACTCACACAC | | TCGTCGAGTTGCTGGTGTAG | | |
| SaChEVE9-t | ACCGGCGCATTTCGTGATTA | | ACTGCCAAATTAGCGCCAAAA | | |
| SgToEVE4-t | CCCGTTCCGTGAAAGAGTGA | | CATACCGGGTCCGAAGAAGG | | |
| SgChEVE4-t | TGCCAGCTGCTAGTTTTAAGC | | TCGGAAAAGGGTACTTGGCA | | |
| SgChEVE34-t | TCCTACAAACCAACCCCACA | | GAAGGACCTGGTTACACCGC | | |
| SgRhEVE7-t | GCAAGGTACACTCCGCTCAT | | TGTGCGTGGGTGATAACGAA | | |
| SgMeEVE4-t | CTCAGTCCTGCGTTGTGGAT | | ACGGCAATCAATAACCTTCCCA | | |
| SgOrEVE6-t | GGATCGCGTTGTGATTTGGG | | AGGAACGAGATGGATGACGC | | |
| SgNyEVE3-t | CAACAGCACCACTTGGTTGTC | | CAGGCCATCTTTCGCGACTT | | |
| RpChEVE8-t | TGCTCTGGACCAAAACTCAAC | | AGTCTGGTTCCTGTAGGCATT | | |
| **Actin Primers used in this study** | | | | |  |
| Sa-Actin | GCGTGACAGGTACATATATACTAA | ACGATTTTGCGTGTCATCCT | |  |  |
| Rp-Actin | GCCCAATCCAAAAGAGGTAT | TCAAAGGTGCTTCCGTTAGT | |  |  |
| Sg-Actin | CGGTTCAAAAACCCAAACCAG | TGGTGATGATTCCCGTGTTC | |  |  |

**CO1 Primers used in this study**

| CO1(Rp) | ATTCAACCAATCATAAAGATATTGG | TAAACTTCTGGATGTCCAAAAAATCA |  |
| --- | --- | --- | --- |
| CO1(Sg) | GGTCAACAAATCATAAAGATATTGG | TAAACTTCAGGGTGACCAAAAAATCA |  |
| ^1^ Sa: *Sitobion avenae*, Rp: *Rhopalosiphum padi*, Sg: *Schizaphis graminum*. -t: transcripts. | | | |
|  | | | |
