# Supplementary material for: Diversity and evolution analysis of RNA viruses in three wheat aphid species
Source: BMC Genomics. 2025 Apr 7;26:353. doi: 10.1186/s12864-025-11512-1 (PMC11978097; doi:10.1186/s12864-025-11512-1)
Supplement: Supplementary file 7 — Suppelemtary Material 7: Table S3. Known ISVs and plant viruses identified in aphid species from the public database [file 12864_2025_11512_MOESM7_ESM.docx]

| **Table S3. Known ISVs and plant viruses identified in aphid species from the public database.** | | | | | | | | | |
| --- | --- | --- | --- | --- | --- | --- | --- | --- | --- |
| **Virus names** | **NCBI Accession** | **Length**  **(nt)** | **Coverage** | **E-value** | **RdRP protein  Identities** | **Virus**  **family** | **RNA type** | **Host** |  |
| Sitobion avenae bunyavirus 1 strain  XY (SaBuV1-XY) | BK068983 | 6313 | 10.6 | 0.0 | 100% | *Phenuiviridae* | -ssRNA | *Sitobion*  *avenae* |  |
| Sitobion avenae iflavirus 1 strain XY (SaIfV1-XY) | BK068969 | 10932 | 1563.1 | 0.0 | 99.64% | *Iflaviridae* | +ssRNA |  |  |
| Broad bean wilt virus 2 strain SA (BBWV2-SA)* | BK069852 | 3437 | 7.2 | 0.0 | 92.00% | *Secoviridae* | +ssRNA |  |  |
| Rhopalosiphum padi virus (RhPV) | QVW10135.1 | 6400 | 37.2 | 0.0 | 99.87% | *Dicistroviridae* | +ssRNA |  |  |
| Dicistroviridae sp. | QJI52003.1 | 8295 | 7.1 | 0.0 | 99.81% | *Dicistroviridae* | +ssRNA |  |  |
| Pyricularia oryzae ourmia-like virus 2 (PoOLV2) | YP_009667033.1 | 2182 | 15.3 | 0.0 | 99.17% | *Botourmiaviridae* | +ssRNA |  |  |
| Magnaporthe oryzae ourmia-like virus strain SA (MoOLV-SA) | BK069853 | 2345 | 46.1 | 0.0 | 92.44% | *Botourmiaviridae* | +ssRNA |  |  |
| Rhopalosiphum padi iflavirus 1 strain BJ (RpIfV1-BJ) | BK068972 | 10150 | 86.7 | 0.0 | 96.40% | *Iflaviridae* | +ssRNA | *Rhopalosiphum padi* |  |
| Rhopalosiphum padi virus (RhPV) | NP_046155.1 | 22692 | 10414.4 | 0.0 | 99.70% | *Dicistroviridae* | +ssRNA |  |  |
| Dicistroviridae sp. | QJI52065.1 | 11599 | 8401.8 | 0.0 | 99.35% | *Dicistroviridae* | +ssRNA |  |  |
| Cripavirus sp. (CpV) | ULF99860.1 | 5884 | 47737.5 | 0.0 | 97.78% | *Dicistroviridae* | +ssRNA |  |  |
| Jingmen bat dicistrovirus 2 (JmBDV-2) | WPV63149.1 | 17056 | 49222.2 | 0.0 | 99.32% | *Dicistroviridae* | +ssRNA |  |  |
| Narcissus degeneration virus (NDV)* | BDD37692.1 | 6346 | 5.7 | 0.0 | 99.76% | *Potyviridae* | +ssRNA |  |  |
| Narcissus yellow stripe virus (NYSV)* | BBE01232.1 | 9252 | 7.6 | 0.0 | 99.37% | *Potyviridae* | +ssRNA |  |  |
| Machlomovirus zeae (MCMV)* | ATY93213.1 | 4399 | 17.4 | 0.0 | 98.76% | *Tombusviridae* | +ssRNA |  |  |
| Totiviridae sp. (TV) | QJI53447.1 | 6418 | 16713.4 | 0.0 | 99.39% | *Totiviridae* | dsRNA |  |  |
| Soybean thrips virus 3 (STV-3) | QPZ88422.1 | 3671 | 6117.9 | 0.0 | 98.26% | unclassified | +ssRNA |  |  |
| Wuhan insect virus 21 (WIV-21) | BBV14765.1 | 3715 | 157.4 | 0.0 | 99.24% | unclassified | +ssRNA |  |  |
| Acyrthosiphon pisum virus (APV) | QAA78869.1 | 9998 | 101.9 | 0.0 | 99.89% | unclassified | +ssRNA |  |  |
| Riboviria sp. | QKN89015.1 | 10004 | 33.3 | 0.0 | 99.46% | unclassified | +ssRNA |  |  |
| Soybean thrips virus 4 (STV-4) | QPZ88419.1 | 6673 | 71788.3 | 0.0 | 99.75% | unclassified | +ssRNA |  |  |
| Barley aphid RNA virus 4 (BARV-4) | UTQ79681.1 | 9105 | 6697.0 | 0.0 | 100.00% | unclassified | +ssRNA |  |  |
| Culex dicistrovirus 2 strain SG (CDV-2-SG) | BK069854 | 10629 | 113328.1 | 0.0 | 88.94% | *Dicistroviridae* | +ssRNA | *Schizaphis graminum* |  |
| Dicistroviridae sp. strain SG (DCV-SG) | BK069855 | 7325 | 89859.4 | 0.0 | 89.31% | *Dicistroviridae* | +ssRNA |  |  |
| Rhopalosiphum padi virus (RhPV) | WPV63149.1 | 16888 | 82579.6 | 0.0 | 99.61% | *Dicistroviridae* | +ssRNA |  |  |
| Jingmen bat dicistrovirus 2 strain SG (JmBDV-2-SG) | BK069856 | 9850 | 30.2 | 0.0 | 86.18% | *Dicistroviridae* | +ssRNA |  |  |
| Jingmen shrew dicistrovirus 1 (JmSDV-1) | WPV63167.1 | 10217 | 475.2 | 0.0 | 99.61% | *Dicistroviridae* | +ssRNA |  |  |
| Barley aphid RNA virus 4 (BAVRV-4) | BBV14754.1 | 9424 | 268.7 | 0.0 | 99.75% | unclassified | +ssRNA |  |  |
| Soybean thrips virus 4 (STV-4) | QPZ88419.1 | 2730 | 17.248024 | 2.96E-35 | 99.75% | unclassified | +ssRNA |  |  |
| Riboviria sp. | QKN89015.1 | 10374 | 195.725172 | 2.93E-104 | 99.5% | unclassified | +ssRNA |  |  |
| Sitobion miscanthi virus 1 (SMV-1) | QCI31816.1 | 9884 | 11.253083 | 0.0 | 100% | unclassified | +ssRNA |  |  |

* Represent plant viruses.
