# Supplementary material for: Diversity and evolution analysis of RNA viruses in three wheat aphid species
Source: BMC Genomics. 2025 Apr 7;26:353. doi: 10.1186/s12864-025-11512-1 (PMC11978097; doi:10.1186/s12864-025-11512-1)
Supplement: Supplementary file 8 — Suppelemtary Material 8: Table S4. Detailed information on viruses used to construct phylogenetic trees [file 12864_2025_11512_MOESM8_ESM.docx]

| **Table S4.** **Detailed information on viruses used to construct phylogenetic trees.** | | | | | | | |  | |  |  |
| --- | --- | --- | --- | --- | --- | --- | --- | --- | --- | --- | --- |
| **Virus name** | **GenBank ID** | **Order** | **family** | **genus** | | | |  | |  |  |
| ***Picornavirales*** |  |  |  | |  |  |  | |  |  |  |
| ***Iflaviridae*** |  |  |  |  | | | |  | |  |  |
| Sitobion avenae ifavirus 1 | PQ181522 | *Picornavirales* | *Iflaviridae* | *Iflavirus* | | | |  | |  |  |
| Sitobion avenae bunyavirus 1 strain XY | BK068969 | *Picornavirales* | *Iflaviridae* | *Iflavirus* | | | |  | |  |  |
| Sitobion avenae iflavirus 2 | BK068970 | *Picornavirales* | *Iflaviridae* | *Iflavirus* | | | |  | |  |  |
| Rhopalosiphum padi iflavirus 1 | BK068971 | *Picornavirales* | *Iflaviridae* | *Iflavirus* | | | |  | |  |  |
| Rhopalosiphum padi iflavirus 1 strain BJ | BK068972 | *Picornavirales* | *Iflaviridae* | *Iflavirus* | | | |  | |  |  |
| Schizaphis graminum ifavirus 1 | BK068973 | *Picornavirales* | *Iflaviridae* | *Iflavirus* | | | |  | |  |  |
| Lymantria dispar iflavirus 1 | AIF75200.1 | *Picornavirales* | *Iflaviridae* | *Iflavirus* | | | |  | |  |  |
| Bombyx mori iflavirus | BAS18834.1 | *Picornavirales* | *Iflaviridae* | *Iflavirus* | | | |  | |  |  |
| Heliconius erato iflavirus | AHW98099.1 | *Picornavirales* | *Iflaviridae* | *Iflavirus* | | | |  | |  |  |
| Varroa destructor virus 1 | AAP51418.2 | *Picornavirales* | *Iflaviridae* | *Iflavirus* | | | |  | |  |  |
| Laodelphax striatella honeydew virus 1 | AHK05791.1 | *Picornavirales* | *Iflaviridae* | Unknow | | | |  | |  |  |
| Brevicoryne brassicae virus | ABP57198.1 | *Picornavirales* | *Iflaviridae* | *Iflavirus* | | | |  | |  |  |
| Graminella nigrifrons virus 1 | AJT58559.1 | *Picornavirales* | *Iflaviridae* | Unknow | | | |  | |  |  |
| Slow bee paralysis virus | ABS84820.1 | *Picornavirales* | *Iflaviridae* | *Iflavirus* | | | |  | |  |  |
| Halyomorpha halys virus | AGY34702.1 | *Picornavirales* | *Iflaviridae* | Unknow | | | |  | |  |  |
| Sacbrood virus | AAD20260.1 | *Picornavirales* | *Iflaviridae* | *Iflavirus* | | | |  | |  |  |
| La Jolla virus | AKH40287.1 | *Picornavirales* | *Iflaviridae* | Unknow | | | |  | |  |  |
| Lygus lineolaris virus 1 | AEL30247.1 | *Picornavirales* | *Iflaviridae* | *Iflavirus* | | | |  | |  |  |
| Ectropis obliqua picorna-like virus | AAQ64627.1 | *Picornavirales* | *Iflaviridae* | *Iflavirus* | | | |  | |  |  |
| Perina nuda virus | AAL06289.1 | *Picornavirales* | *Iflaviridae* | *Iflavirus* | | | |  | |  |  |
| Spodoptera exigua iflavirus 2 | AHX00961.1 | *Picornavirales* | *Iflaviridae* | *Iflavirus* | | | |  | |  |  |
| Infectious flacherie virus | BAA25371.1 | *Picornavirales* | *Iflaviridae* | *Iflavirus* | | | |  | |  |  |
| Armigeres iflavirus | YP_009448183.1 | *Picornavirales* | *Iflaviridae* | *Iflavirus* | | | |  | |  |  |
| Psammotettix alienus iflavirus 1 | AYD38337.1 | *Picornavirales* | *Iflaviridae* | *Iflavirus* | | | |  | |  |  |
| Nilaparvata lugens honeydew virus-2 | BAN57352.1 | *Picornavirales* | *Iflaviridae* | Unknow | | | |  | |  |  |
| Nilaparvata lugens honeydew virus-3 | BAN57353.1 | *Picornavirales* | *Iflaviridae* | Unknow | | | |  | |  |  |
| Tribolium castaneum iflavirus | WIF15501.1 | *Picornavirales* | *Iflaviridae* | Unknow | | | |  | |  |  |
| Nilaparvata lugens honeydew virus 1 | YP_009505599.1 | *Picornavirales* | *Iflaviridae* | *Iflavirus* | | | |  | |  |  |
| Spodoptera exigua iflavirus 1 | YP_004935363.1 | *Picornavirales* | *Iflaviridae* | *Iflavirus* | | | |  | |  |  |
| Pityohyphantes rubrofasciatus iflavirus | YP_009351892.1 | *Picornavirales* | *Iflaviridae* | Unknow | | | |  | |  |  |
| Wufeng shrew iflavirus 6 | WPV63248.1 | *Picornavirales* | *Iflaviridae* | *Iflavirus* | | | |  | |  |  |
| Iflaviridae sp. | QKN89074.1 | *Picornavirales* | *Iflaviridae* | Unknow | | | |  | |  |  |
| Spodoptera exigua virus | AHX00963.1 | *Picornavirales* | *Picornaviridae* | Unknow | | | |  | |  |  |
| Drosophila immigrans Nora virus | YP_009047190.1 | unclassified |  |  | | | |  | |  |  |
| ***Solinviviridae*** |  |  |  |  | | | |  | |  |  |
| Rhopalosiphum padi Solinvi-like virus 1 | BK068974 | *Picornavirales* | *Solinviviridae* | Unknow | | | |  | |  |  |
| Leptinotarsa solinvi-like virus 1 | WNV56444.1 | *Picornavirales* | *Solinviviridae* | Unknow | | | |  | |  |  |
| Nylanderia fulva virus 1 | ANQ44728.1 | *Picornavirales* | *Solinviviridae* | *Nyfulvavirus* | | | |  | |  |  |
| Solenopsis invicta virus 3 | ACO37271.1 | *Picornavirales* | *Solinviviridae* | *Invictavirus* | | | |  | |  |  |
| Fushun Solinvi-like virus 1 | UHM27685.1 | *Picornavirales* | *Solinviviridae* | Unknow | | | |  | |  |  |
| Sanya ochthera mantis Solinvi-like virus 1 | UHM27575.1 | *Picornavirales* | *Solinviviridae* | Unknow | | | |  | |  |  |
| Sanya gryllotalpa orientalis Solinvi-like virus 1 | UHK03114.1 | *Picornavirales* | *Solinviviridae* | Unknow | | | |  | |  |  |
| Hangzhou scatella stagnalis Solinvi-like virus 1 | UHR49739.1 | *Picornavirales* | *Solinviviridae* | Unknow | | | |  | |  |  |
| Macrobrachium rosenbergii virus 1 | UUV42128.1 | *Picornavirales* | *Solinviviridae* | Unknow | | | |  | |  |  |
| Penaeus vannamei solinvivirus | UWY63979.1 | *Picornavirales* | *Solinviviridae* | Unknow | | | |  | |  |  |
| Sanya orius sauteri Solinvi-like virus 1 | UHR49807.1 | *Picornavirales* | *Solinviviridae* | Unknow | | | |  | |  |  |
| Hangzhou Solinvi-like virus 1 | UHR49784.1 | *Picornavirales* | *Solinviviridae* | Unknow | | | |  | |  |  |
| Lasius neglectus picorna-like virus 3 | UXD80005.1 | *Picornavirales.* | *Picornaviridae* | Unknow | | | |  | |  |  |
| Wufeng shrew picorna-like virus 51 | WPV63590.1 | *Picornavirales.* | *Picornaviridae* | Unknow | | | |  | |  |  |
| Ixodes ricinus picorna-like virus 1 | USL85443.1 | *Picornavirales.* | *Picornaviridae* | Unknow | | | |  | |  |  |
| Areca palm necrotic ringspot virus | UIW31246.1 | *Patatavirales* | *Potyviridae* | *Arepavirus* | | | |  | |  |  |
| ***Dicistroviridae*** |  |  |  |  | | | |  | |  |  |
| Dicistroviridae sp. | UGO57581.1 | *Picornavirales* | *Dicistroviridae* | Unknow | | | |  | |  |  |
| Shenzhen dicistro-like virus | QPN36927.1 | *Picornavirales* | *Dicistroviridae* | Unknow | | | |  | |  |  |
| Bivalve RNA virus G5 | QUS52502.1 | *Picornavirales* | *Dicistroviridae* | Unknow | | | |  | |  |  |
| Aparavirus sp. | ULF50555.1 | *Picornavirales* | *Dicistroviridae* | *Aparavirus* | | | |  | |  |  |
| Human blood-associated dicistrovirus | AWK23470.1 | *Picornavirales* | *Dicistroviridae* | Unknow | | | |  | |  |  |
| Taura syndrome virus | NP_149057.1 | *Picornavirales* | *Dicistroviridae* | *Aparavirus* | | | |  | |  |  |
| Triatoma virus | NP_620562.1 | *Picornavirales* | *Dicistroviridae* | *Triatovirus* | | | |  | |  |  |
| Cricket paralysis virus | NP_647481.1 | *Picornavirales* | *Dicistroviridae* | *Cripavirus* | | | |  | |  |  |
| ***Marnaviridae*** |  |  |  |  | | | |  | |  |  |
| Marnaviridae sp. | ULF99966.1 | *Picornavirales* | *Marnaviridae* | Unknow | | | |  | |  |  |
| Macrobrachium rosenbergii virus 5 | UUV42145.1 | *Picornavirales* | *Marnaviridae* | *Sogarnavirus* | | | |  | |  |  |
| ***Picornaviridae*** |  |  |  |  | | | |  | |  |  |
| Fur seal picorna-like virus | YP_009388485.1 | *Picornavirales* | *Picornaviridae* | Unknow | | | |  | |  |  |
| Freshwater macrophyte associated picorna-like virus 15 | UQZ09577.1 | *Picornavirales* | *Picornaviridae* | Unknow | | | |  | |  |  |
| Parechovirus A | NP_046804.1 | *Picornavirales* | *Picornaviridae* | *Parechovirus* | | | |  | |  |  |
| Enterovirus C | NP_041277.1 | *Picornavirales* | *Picornaviridae* | *Enterovirus* | | | |  | |  |  |
| aichivirus A1 | NP_047200.1 | *Picornavirales* | *Picornaviridae* | *Kobuvirus* | | | |  | |  |  |
| Leptasterias associated picornavirus 1 | DAZ87482.1 | *Picornavirales* | *Picornaviridae* | Unknow | | | |  | |  |  |
| Apostichopus japonicus associated picornavirus 1 | DAZ87472.1 | *Picornavirales* | *Picornaviridae* | Unknow | | | |  | |  |  |
| ***Secoviridae*** |  |  |  |  | | | |  | |  |  |
| Arabis mosaic virus | YP_053925.1 | *Picornavirales* | *Secoviridae* | *Nepovirus* | | | |  | |  |  |
| Tomato torrado virus | YP_001039627.1 | *Picornavirales* | *Secoviridae* | *Torradovirus* | | | |  | |  |  |
| Strawberry mottle virus | NP_599086.1 | *Picornavirales* | *Secoviridae* | *Sadwavirus* | | | |  | |  |  |
| Parsnip yellow fleck virus | NP_619734.1 | *Picornavirales* | *Secoviridae* | *Sequivirus* | | | |  | |  |  |
| Rice tungro spherical virus | NP_042507.1 | *Picornavirales* | *Secoviridae* | *Waikavirus* | | | |  | |  |  |
| ***Polycipiviridae*** |  |  |  |  | | | |  | |  |  |
| Polycipiviridae sp. | AZL87720.1 | *Picornavirales* | *Polycipiviridae* | Unknow | | | |  | |  |  |
| Hubei picorna-like virus 82 | YP_009330058 | *Picornavirales* | *Polycipiviridae* | *Chipolycivirus* | | | |  | |  |  |
| Lasius niger virus 1 | UXD80070.1 | *Picornavirales* | *Polycipiviridae* | *Sopolycivirus* | | | |  | |  |  |
| Solenopsis invicta virus 2 | ASK12217.1 | *Picornavirales* | *Polycipiviridae* | *Sopolycivirus* | | | |  | |  |  |
| Picornavirales sp. | UNY41981.1 | *Picornavirales* | Unknow |  | | | |  | |  |  |
| Picornavirales Q_sR_OV_025 | ASG92552.1 | *Picornavirales* | Unknow |  | | | |  | |  |  |
| Nelson Picorna-like virus 3 | QZZ63320.1 | *Picornavirales* | Unknow |  | | | |  | |  |  |
| Macrobrachium rosenbergii virus 12 | UUV42131.1 | *Picornavirales* | Unknow |  | | | |  | |  |  |
| Ginkgo biloba picorna-like virus | QKK82972.1 | *Picornavirales* | Unknow |  | | | |  | |  |  |
| Trichosanthes kirilowii picorna-like virus | QKK82970.1 | *Picornavirales* | Unknow |  | | | |  | |  |  |
| Lasius neglectus picorna-like virus 4 | UXD80095.1 | *Picornavirales* | Unknow |  | | | |  | |  |  |
| ***Mononegavirales Lispiviridae*** |  |  |  |  | | | |  | |  |  |
| Sitobion avenae lispivirus 1 | BK068975 | *Mononegavirales* | *Lispiviridae* | *Arlivirus* | | | |  | |  |  |
| Guiyang lispivirus 1 | YP_010805224.1 | *Mononegavirales* | *Lispiviridae* | Unknow | | | |  | |  |  |
| Arlivirus sp. | YP_010805183.1 | *Mononegavirales* | *Lispiviridae* | *Arlivirus* | | | |  | |  |  |
| Bemisia tabaci arlivirus 2 | YP_010804980.1 | *Mononegavirales* | *Lispiviridae* | *Arlivirus* | | | |  | |  |  |
| Bemisia tabaci arlivirus 1 | YP_010804974.1 | *Mononegavirales* | *Lispiviridae* | *Arlivirus* | | | |  | |  |  |
| Leveillula taurica associated rhabdo-like virus 1 | QLL27736.1 | *Mononegavirales* | *Rhabdoviridae* | Unknow | | | |  | |  |  |
| Sanxia water strider virus 4 | YP_009288955.1 | *Mononegavirales* | *Lispiviridae* | *Sanstrivirus* | | | |  | |  |  |
| Hangzhou eysarcoris guttigerus lispivirus 1 | YP_010805207.1 | *Mononegavirales* | *Lispiviridae* | Unknow | | | |  | |  |  |
| Hubei odonate virus 10 | YP_009336924.1 | *Mononegavirales* | *Lispiviridae* | *Damravirus* | | | |  | |  |  |
| Hemipteran arli-related virus OKIAV94 | QMP82354.1 | *Mononegavirales* | *Lispiviridae* | *Rivapovirus* | | | |  | |  |  |
| Hemipteran arli-related virus OKIAV95 | QPL15300.1 | *Mononegavirales* | *Lispiviridae* | *Xenophyvirus* | | | |  | |  |  |
| Lishi Spider Virus 2 | AJG39111.1 | *Mononegavirales* | *Lispiviridae* | *Arlivirus* | | | |  | |  |  |
| Tacheng Tick Virus 6 | YP_009304420.1 | *Mononegavirales* | *Lispiviridae* | *Ganiavirus* | | | |  | |  |  |
| Wuchang romanomermis nematode virus 2 | YP_009342285.1 | *Mononegavirales* | *Lispiviridae* | *Nematovirus* | | | |  | |  |  |
| Amsterdam virus | QEQ50497.1 | *Mononegavirales* | *Lispiviridae* | *Usmuvirus* | | | |  | |  |  |
| Coleopteran arli-related virus OKIAV107 | QMP82314.1 | *Mononegavirales* | *Lispiviridae* | *Cybitervirus* | | | |  | |  |  |
| Megalopteran arli-related virus OKIAV106 | QMP82230.1 | *Mononegavirales* | *Lispiviridae* | *Arlivirus* | | | |  | |  |  |
| Hubei rhabdo-like virus 3 | YP_009336889.1 | *Mononegavirales* | *Lispiviridae* | *Leocovirus* | | | |  | |  |  |
| Strepsipteran arli-related virus OKIAV104 | QMP82292.1 | *Mononegavirales* | *Lispiviridae* | *Stylovirus* | | | |  | |  |  |
| Blattodean arli-related virus OKIAV101 | QMP82176.1 | *Mononegavirales* | *Lispiviridae* | *Arlivirus* | | | |  | |  |  |
| Blattodean arli-related virus OKIAV102 | QMP82242.1 | *Mononegavirales* | *Lispiviridae* | *Supelovirus* | | | |  | |  |  |
| Jimsystermes virus | QQM16270.1 | *Mononegavirales* | *Lispiviridae* | *Copasivirus* | | | |  | |  |  |
| Isopteran arli-related virus OKIAV103 | QMP82349.1 | *Mononegavirales* | *Lispiviridae* | *Copasivirus* | | | |  | |  |  |
| Hymenopteran arli-related virus OKIAV98 | QPL15295.1 | *Mononegavirales* | *Lispiviridae* | *Synelinevirus* | | | |  | |  |  |
| Linepithema humile rhabdo-like virus 1 | AXA52562.1 | *Mononegavirales* | *Lispiviridae* | *Synelinevirus* | | | |  | |  |  |
| Anisopteromalus calandrae negative-strand RNA virus 2 | QWT43291.1 | *Mononegavirales* | *Lispiviridae* | *Anicalvirus* | | | |  | |  |  |
| Anisopteromalus calandrae negative-strand RNA virus 1 | QWT43285.1 | *Mononegavirales* | *Lispiviridae* | *Anidravirus* | | | |  | |  |  |
| Hymenopteran arli-related virus OKIAV99 | QPL15345.1 | *Mononegavirales* | *Lispiviridae* | *Phelinovirus* | | | |  | |  |  |
| Beihai rhabdo-like virus 6 | APG78639.1 | *Mononegavirales* | *Nyamiviridae* | *Crustavirus* | | | |  | |  |  |
| **Negevirus** |  |  |  |  | | | |  | |  |  |
| Sitobion avenae nege-like virus 1 | BK068977 |  | Unknow |  | | | |  | |  |  |
| Loreto virus | YP_009351835.1 |  |  | Negevirus | | | |  | |  |  |
| Big Cypress virus | YP_009351821.1 |  | unclassified |  | | | |  | |  |  |
| Ying Kou virus | YP_009552739.1 |  |  | Negevirus | | | |  | |  |  |
| Negevirus Nona 1 | BAS69360.1 |  |  | Negevirus | | | |  | |  |  |
| Daeseongdong virus 1 | YP_009182191.1 |  | unclassified |  | | | |  | |  |  |
| Piura virus | YP_009351830.1 |  |  | Negevirus | | | |  | |  |  |
| Castlerea virus | YP_009362298.1 |  |  | Negevirus | | | |  | |  |  |
| West Accra virus | BBN20799.1 |  | unclassified |  | | | |  | |  |  |
| Manglie virus | QBR99594.1 |  | unclassified |  | | | |  | |  |  |
| Negev virus | YP_009256205.1 |  |  | Negevirus | | | |  | |  |  |
| Barley aphid RNA virus 1 | BBV14745.1 |  | unclassified |  | | | |  | |  |  |
| Astegopteryx formosana nege-like virus 1 | URA30365.1 |  |  | Negevirus | | | |  | |  |  |
| Indomegoura indica nege-like virus 1 | QUE41594.1 |  | unclassified |  | | | |  | |  |  |
| Wuhan insect virus 8 | YP_009344994.1 |  | unclassified |  | | | |  | |  |  |
| Wuhan house centipede virus 1 | YP_009342435.1 |  | unclassified |  | | | |  | |  |  |
| Aphis glycines nege-like virus 1 | UTQ79653.1 |  | unclassified |  | | | |  | |  |  |
| Citrus leprosis virus C | ABC75821.1 | *Martellivirales* | *Kitaviridae* | *Cilevirus* | | | |  | |  |  |
| ***Bunyavirales Phenuiviridae*** |  |  |  |  | | | |  | |  |  |
| Sitobion avenae bunyavirus 1 | BK068982 | *Bunyavirales* | *Phenuiviridae* | *Citricidus* | | | |  | |  |  |
| Sitobion avenae bunyavirus 1 strain XY | BK068983 | *Bunyavirales* | *Phenuiviridae* | *Citricidus* | | | |  | |  |  |
| Aphis citricidus bunyavirus | YP_010840741.1 | *Bunyavirales* | *Phenuiviridae* | *Citricidus* | | | |  | |  |  |
| Blacklegged tick phlebovirus 3 | ANC97695.1 | *Bunyavirales* | *Phenuiviridae* | *Ixovirus* | | | |  | |  |  |
| Blacklegged tick phlebovirus 2 | AII01807.1 | *Bunyavirales* | *Phenuiviridae* | *Phlebovirus* | | | |  | |  |  |
| Razdan virus | AGY30953.1 | *Bunyavirales* | *Phenuiviridae* | *Bandavirus* | | | |  | |  |  |
| Yongjia Tick Virus 1 | YP_010086231.1 | *Bunyavirales* | *Phenuiviridae* | *Uukuvirus* | | | |  | |  |  |
| Tongren Phenu tick virus 1 | UYL95523.1 | *Bunyavirales* | *Phenuiviridae* | Unknow | | | |  | |  |  |
| Phenuiviridae sp. | WAK75710.1 | *Bunyavirales* | *Phenuiviridae* | Unknow | | | |  | |  |  |
| Huangpi Tick Virus 2 | YP_009293590.1 | *Bunyavirales* | *Phenuiviridae* | *Phlebovirus* | | | |  | |  |  |
| Tongren Perib tick virus 2 | UYL95521.1 | *Bunyavirales* | *Peribunyaviridae* | Unknow | | | |  | |  |  |
| Mourilyan virus | QPN53433.1 | *Bunyavirales* | *Phenuiviridae* | *Wenrivirus* | | | |  | |  |  |
| Hubi lepidoptera virus 1 | APG79261.1 | *Bunyavirales* | *Phenuiviridae* | *Hudovirus* | | | |  | |  |  |
| Pidgey virus | AOX47534.1 | *Bunyavirales* | *Phenuiviridae* | *Pidchovirus* | | | |  | |  |  |
| Habi diptera virus 4 | APG79298.1 | *Bunyavirales* | *Phenuiviridae* | *Hudivirus* | | | |  | |  |  |
| Hubi diptera virus 3 | APG79285.1 | *Bunyavirales* | *Phenuiviridae* | *Beidivirus* | | | |  | |  |  |
| Guadeloupe mosquito phasivirus | QEM39249.1 | *Bunyavirales* | *Phenuiviridae* | *Phasivirus* | | | |  | |  |  |
| Dar es Salaam virus | QDF82060.1 | *Bunyavirales* | *Phenuiviridae* | *Tanzavirus* | | | |  | |  |  |
| Wuhn horsefly virus | AJG39260.1 | *Bunyavirales* | *Phenuiviridae* | *Horwuvirus* | | | |  | |  |  |
| melon chlorotic spot virus | AYL40766.1 | *Bunyavirales* | *Phenuiviridae* | *Mechlorovirus* | | | |  | |  |  |
| European wheat striate mosaic virus | YP_010840102.1 | *Bunyavirales* | *Phenuiviridae* | *Tenuivirus* | | | |  | |  |  |
| Gouleako virus | AEJ38175.1 | *Bunyavirales* | *Phenuiviridae* | *Goukovirus* | | | |  | |  |  |
| Mothra virus | AOF41426.1 | *Bunyavirales* | *Phenuiviridae* | *Mobuvirus* | | | |  | |  |  |
| apple rubbery wood virus 1 | AWC67511.1 | *Bunyavirales* | *Phenuiviridae* | *Rubodvirus* | | | |  | |  |  |
| Entoleuca phenui-like virus 1 | YP_010086241.1 | *Bunyavirales* | *Phenuiviridae* | *Entovirus* | | | |  | |  |  |
| Lentinula edodes negative-strand RNA virus 2 | YP_010086265.1 | *Bunyavirales* | *Phenuiviridae* | *Lentinuvirus* | | | |  | |  |  |
| Laurel Lake virus | ASU47549.1 | *Bunyavirales* | *Phenuiviridae* | *Laulavirus* | | | |  | |  |  |
| Citrus concave gum-associated virus | AST13127.1 | *Bunyavirales* | *Phenuiviridae* | *Coguvirus* | | | |  | |  |  |
| Issyk-Kul virus | WOE94926.1 | *Bunyavirales* | *Nairoviridae* | *Orthonairovirus* | | | |  | |  |  |
| ***Tolivirales Tombusviridae*** |  |  |  |  | | | |  | |  |  |
| Sitobion avenae tombus-like virus 1 | BK068978 |  | unclassified |  | | | |  | |  |  |
| Changjiang tombus-like virus 8 | YP_009337198.1 |  | unclassified |  | | | |  | |  |  |
| Wenling tombus-like virus 1 | YP_009337158.1 |  | unclassified |  | | | |  | |  |  |
| Hubei tombus-like virus 11 | YP_009336956.1 |  | unclassified |  | | | |  | |  |  |
| Beihai tombus-like virus 8 | YP_009336938.1 |  | unclassified |  | | | |  | |  |  |
| Beihai tombus-like virus 1 | YP_009336751.1 |  | unclassified |  | | | |  | |  |  |
| Hubei tombus-like virus 12 | YP_009336735.1 |  | unclassified |  | | | |  | |  |  |
| Sclerotinia sclerotiorum umbra-like virus 1 | YP_009253998.1 | *Tolivirales* | *Tombusviridae* | *Umbravirus* | | | |  | |  |  |
| Rosa rugosa leaf distortion virus | YP_007501034.1 | *Tolivirales* | *Tombusviridae* | *Pelarspovirus* | | | |  | |  |  |
| Tobacco bushy top virus | NP_733848.2 | *Tolivirales* | *Tombusviridae* | *Umbravirus* | | | |  | |  |  |
| Turnip crinkle virus | NP_620720.3 | *Tolivirales* | *Tombusviridae* | *Betacarmovirus* | | | |  | |  |  |
| Tomato bushy stunt virus | NP_062897.1 | *Tolivirales* | *Tombusviridae* | *Tombusvirus* | | | |  | |  |  |
| Diaporthe ambigua RNA virus 1 | NP_037581.1 |  | unclassified |  | | | |  | |  |  |
| Verticillium dahliae RNA virus | AQM49992.1 |  | unclassified |  | | | |  | |  |  |
| Wenzhou tombus-like virus 5 | APG76630.1 |  | unclassified |  | | | |  | |  |  |
| Beihai tombus-like virus 6 | APG76145.1 |  | unclassified |  | | | |  | |  |  |
| Soybean leaf-associated ssRNA virus 3 | ALM62246.1 |  | unclassified |  | | | |  | |  |  |
| Soybean leaf-associated ssRNA virus 2 | ALM62236.1 |  | unclassified |  | | | |  | |  |  |
| Soybean leaf-associated ssRNA virus 1 | ALM62232.1 |  | unclassified |  | | | |  | |  |  |
| Macrophomina phaseolina single-stranded RNA virus 3 | ALD89104.2 |  | unclassified |  | | | |  | |  |  |
| Magnaporthe oryzae RNA virus | AJA41112.1 |  | unclassified |  | | | |  | |  |  |
| Rose yellow leaf virus | AGF70700.1 | *Tolivirales* | *Tombusviridae* |  | | | |  | |  |  |
| Trailing lespedeza virus 1 | ADY69093.2 | *Tolivirales* | *Tombusviridae* | *Tralespevirus* | | | |  | |  |  |
| Carrot mottle mimic virus | ACJ03572.1 | *Tolivirales* | *Tombusviridae* | *Umbravirus* | | | |  | |  |  |
| Maize chlorotic mottle virus | ACA57840.1 | *Tolivirales* | *Tombusviridae* | *Machlomovirus* | | | |  | |  |  |
| Pea enation mosaic virus 2 | AAU20330.2 | *Tolivirales* | *Tombusviridae* | *Umbravirus* | | | |  | |  |  |
| Cowpea mottle virus | AAC54603.1 | *Tolivirales* | *Tombusviridae* | *Gammacarmovirus* | | | |  | |  |  |
| Diplodia fraxini fusagravirus 1b | WNT71265.1 |  |  |  | | | |  | |  |  |
| ***Mononegavirales Rhabdoviridae*** |  |  |  |  | | | |  | |  |  |
| Rhopalosiphum padi rhabdo-like virus 1 | BK068979 | *Mononegavirales* | *Rhabdoviridae* | *Almendravirus* | | | |  | |  |  |
| Blanchseco virus | QDW81033.1 | *Mononegavirales* | *Rhabdoviridae* | *Alpharicinrhavirus* | | | |  | |  |  |
| Rattus tanezumi rhabdovirus 1 | QIM74105.1 | *Mononegavirales* | *Rhabdoviridae* | *Alphanemrhavirus* | | | |  | |  |  |
| Puerto Almendras virus | AHU86506.1 | *Mononegavirales* | *Rhabdoviridae* | *Almendravirus* | | | |  | |  |  |
| Frog lyssa-like virus 1 | QCF24331.1 | *Mononegavirales* | *Rhabdoviridae* | *Amplylivirus* | | | |  | |  |  |
| Lyssavirus aravan] | ABV03822.1 | *Mononegavirales* | *Rhabdoviridae* | *Lyssavirus* | | | |  | |  |  |
| Anole lyssa-like virus 1 | FAA01392.1 | *Mononegavirales* | *Rhabdoviridae* | *Replylivirus* | | | |  | |  |  |
| Connecticut virus | AJR28559.1 | *Mononegavirales* | *Rhabdoviridae* | *Sawgrhavirus* | | | |  | |  |  |
| Moussa virus | ACZ81402.1 | *Mononegavirales* | *Rhabdoviridae* | *Mousrhavirus* | | | |  | |  |  |
| Bahia Grande virus | AJR28545.1 | *Mononegavirales* | *Rhabdoviridae* | *Barhavirus* | | | |  | |  |  |
| Zahedan rhabdovirus | AJR16768.1 | *Mononegavirales* | *Rhabdoviridae* | *Zarhavirus* | | | |  | |  |  |
| Xiniiang tick rhabdovirus | QBQ65046.1 | *Mononegavirales* | *Rhabdoviridae* | *Lostrhavirus* | | | |  | |  |  |
| Solenopsis invicta virus 3 | ACO37271 | *Picornavirales* | *Solinviviridae* |  | | | |  | |  |  |
| ***Wolframvirales Narnaviridae*** |  |  |  |  | | | |  | |  |  |
| Rhopalosiphum padi narna-like virus 1 | BK068980 | *Wolframvirales* | *Narnaviridae* | Unknow | | | |  | |  |  |
| Ustilaginoidea virens narnavirus 1 | WKF54344.1 | *Wolframvirales* | *Narnaviridae* | *Narnavirus* | | | |  | |  |  |
| Streptophyte associated narna-like virus 9 | WPR17282.1 | *Wolframvirales* | *Narnaviridae* | Unknow | | | |  | |  |  |
| Aspergillus fumigatus narnavirus 1 | AXE72933 | *Wolframvirales* | *Narnaviridae* | *Narnavirus* | | | |  | |  |  |
| Plasmopara viticola lesion associated narnavirus 11 | QIR30290 | *Wolframvirales* | *Narnaviridae* | Unknow | | | |  | |  |  |
| Neofusicoccum parvum narnavirus 1 | QDB74994 | *Wolframvirales* | *Narnaviridae* | Unknow | | | |  | |  |  |
| Culex narnavirus 1 | QTJ62749.1 | *Wolframvirales* | *Narnaviridae* | Unknow | | | |  | |  |  |
| Xanthi narna-like virus | QRD99904 | *Wolframvirales* | *Narnaviridae* | Unknow | | | |  | |  |  |
| Soybean thrips narna-like virus 1 | QQP18719 | *Wolframvirales* | *Narnaviridae* | Unknow | | | |  | |  |  |
| Aedes japonicus narnavirus 1 | BCI50700 | *Wolframvirales* | *Narnaviridae* | Unknow | | | |  | |  |  |
| Insect narna-like virus 2 | QNM37824 | *Wolframvirales* | *Narnaviridae* | Unknow | | | |  | |  |  |
| Alternaria alternata mitovirus 1 | QDB74990.1 | *Cryppavirales* | *Mitoviridae* | *Duamitoviru* | | | |  | |  |  |
| ***Durnavirales Fusariviridae*** |  |  |  |  | | | |  | |  |  |
| Rhopalosiphum padi fusarivirus 1 | BK068981 | *Durnavirales* | *Fusariviridae* | *Alphafusarivirus* | | | |  | |  |  |
| Agaricus bisporus virus 10 | AQM49936 | *Durnavirales* | *Fusariviridae* | *Alphafusarivirus* | | | |  | |  |  |
| Aspergillus ellipticus fusarivirus 1 | AZT88650.1 | *Durnavirales* | *Fusariviridae* | *Alphafusarivirus* | | | |  | |  |  |
| Auricularia heimuer fusarivirus 1 | QJP04102.1 | *Durnavirales* | *Fusariviridae* | *Alphafusarivirus* | | | |  | |  |  |
| Botryosphaeria dothidea fusarivirus 1 | QHI00151.1 | *Durnavirales* | *Fusariviridae* | *Alphafusarivirus* | | | |  | |  |  |
| Alternaria brassicicola fusarivirus 1 | ALW95411.1 | *Durnavirales* | *Fusariviridae* | *Betafusarivirus* | | | |  | |  |  |
| Botrytis cinerea fusarivirus 7 | QJT73723.1 | *Durnavirales* | *Fusariviridae* | *Betafusarivirus* | | | |  | |  |  |
| Rhizoctonia solani fusarivirus 2 | QDW92691.1 | *Durnavirales* | *Fusariviridae* | *Betafusarivirus* | | | |  | |  |  |
| Sclerotinia homoeocarpa fusarivirus 1 | AZT88661.1 | *Durnavirales* | *Fusariviridae* | *Betafusarivirus* | | | |  | |  |  |
| Lentinula edodes fusarivirus 1 | QOX06044.1 | *Durnavirales* | *Fusariviridae* | *Gammafusarivirus* | | | |  | |  |  |
| Phomopsis vexans fusarivirus 1 | QZW16103.1 | *Durnavirales* | *Fusariviridae* | Unknow | | | |  | |  |  |
| Penicillium roqueforti ssRNA mycovirus 1 | YP_009052456 | *Durnavirales* | *Fusariviridae* | *Alphafusarivirus* | | | |  | |  |  |
| Fusariviridae sp. | WAK72339 | *Durnavirales* | *Fusariviridae* | Unknow | | | |  | |  |  |
| Trichoderma harzianum hypovirus 1 | QGA30970.1 | *Durnavirales* | *Hypoviridae* | *Hypovirus* | | | |  | |  |  |

**Details of sequences obtained from GenBank for the construction of EVE phylogenetic trees.**

***Durnavirales Partitiviridae***

| Beet cryptic virus 1 | NC_011556 | *Durnavirales* | *Partitiviridae* | *Alphapartitivirus* |
| --- | --- | --- | --- | --- |
| Carrot cryptic virus | NC_038824 | *Durnavirales* | *Partitiviridae* | *Alphapartitivirus* |
| Amasya cherry disease-associated mycovirus | NC_006441 | *Durnavirales* | *Partitiviridae* | *Alphapartitivirus* |
| Chondrostereum purpureum cryptic virus 1 | NC_038916 | *Durnavirales* | *Partitiviridae* | *Alphapartitivirus* |
| Flammulina velutipes browning virus | NC_038826 | *Durnavirales* | *Partitiviridae* | *Alphapartitivirus* |
| Helicobasidium mompa dsRNA mycovirus | NC_043392 | *Durnavirales* | *Partitiviridae* | *Alphapartitivirus* |
| Heterobasidion partitivirus 1 | NC_038827 | *Durnavirales* | *Partitiviridae* | *Alphapartitivirus* |
| Rosellinia necatrix partitivirus 2 | NC_020234 | *Durnavirales* | *Partitiviridae* | *Alphapartitivirus* |
| Vicia cryptic virus | NC_007241 | *Durnavirales* | *Partitiviridae* | *Alphapartitivirus* |
| White clover cryptic virus 1 | NC_006275 | *Durnavirales* | *Partitiviridae* | *Alphapartitivirus* |
| Atkinsonella hypoxylon virus | NC_003470 | *Durnavirales* | *Partitiviridae* | *Betapartitivirus* |
| Cannabis cryptic virus | NC_031134 | *Durnavirales* | *Partitiviridae* | *Betapartitivirus* |
| Ceratocystis resinifera virus 1 | NC_010755 | *Durnavirales* | *Partitiviridae* | *Betapartitivirus* |
| Ceratocystis polonica partitivirus | NC_010705 | *Durnavirales* | *Partitiviridae* | *Betapartitivirus* |
| Crimson clover cryptic virus 2 | NC_038837 | *Durnavirales* | *Partitiviridae* | *Betapartitivirus* |
| Dill cryptic virus 2 | NC_021147 | *Durnavirales* | *Partitiviridae* | *Betapartitivirus* |
| Fusarium poae virus 1 | NC_003884 | *Durnavirales* | *Partitiviridae* | *Betapartitivirus* |
| Heterobasidion partitivirus 2 | NC_038839 | *Durnavirales* | *Partitiviridae* | *Betapartitivirus* |
| Hop trefoil cryptic virus 2 | NC_021098 | *Durnavirales* | *Partitiviridae* | *Betapartitivirus* |
| Pleurotus ostreatus virus 1 | NC_006961 | *Durnavirales* | *Partitiviridae* | *Betapartitivirus* |
| Red clover cryptic virus 2 | NC_021096 | *Durnavirales* | *Partitiviridae* | *Betapartitivirus* |
| White clover cryptic virus 2 | NC_021094 | *Durnavirales* | *Partitiviridae* | *Betapartitivirus* |
| Cryptosporidium parvum virus 1 | NC_038843 | *Durnavirales* | *Partitiviridae* | *Cryspovirus* |
| Beet cryptic virus 2 | NC_038845 | *Durnavirales* | *Partitiviridae* | *Deltapartitivirus* |
| Fig cryptic virus | NC_015494 | *Durnavirales* | *Partitiviridae* | *Deltapartitivirus* |
| Pepper cryptic virus 1 | NC_037095 | *Durnavirales* | *Partitiviridae* | *Deltapartitivirus* |
| Pepper cryptic virus 2 | NC_034159 | *Durnavirales* | *Partitiviridae* | *Deltapartitivirus* |
| Aspergillus ochraceous virus | NC_043396 | *Durnavirales* | *Partitiviridae* | *Gammapartitivirus* |
| Discula destructiva virus 1 | NC_002797 | *Durnavirales* | *Partitiviridae* | *Gammapartitivirus* |
| Discula destructiva virus 2 | NC_003710 | *Durnavirales* | *Partitiviridae* | *Gammapartitivirus* |
| Fusarium solani virus 1 | NC_003885 | *Durnavirales* | *Partitiviridae* | *Gammapartitivirus* |
| Gremmeniella abietina RNA virus MS1 | NC_004018 | *Durnavirales* | *Partitiviridae* | *Gammapartitivirus* |
| Ophiostoma partitivirus 1 | NC_038918 | *Durnavirales* | *Partitiviridae* | *Gammapartitivirus* |
| Penicillium stoloniferum virus F | NC_007221 | *Durnavirales* | *Partitiviridae* | *Gammapartitivirus* |
| Penicillium stoloniferum virus S | NC_005976 | *Durnavirales* | *Partitiviridae* | *Gammapartitivirus* |
| Hubei partiti-like virus 56 | APG78242.1 | unclassified |  |  |
| Chihuahua culicoides partitivirus 1 | XDO01458.1 | *Durnavirales* | *Partitiviridae* | Unknow |
| Changjiang partiti-like virus 1 | APG78201.1 | unclassified |  |  |
| XiangYun partiti-picobirna-like virus 4 | UUG74126.1 | unclassified |  |  |
| Culex pseudovishnui partitivirus | BBQ05103.1 | *Durnavirales* | *Partitiviridae* |  |
| Vaasa deltapartitivirus | UUV42389.1 | *Durnavirales* | *Partitiviridae* | *Deltapartitivirus* |
| Ilomantsi deltapartitivirus | UUV42360.1 | *Durnavirales* | *Partitiviridae* | *Deltapartitivirus* |
| Inari deltapartitivirus | UUV42371.1 | *Durnavirales* | *Partitiviridae* | *Deltapartitivirus;* |
| Hubei partiti-like virus 57 | APG78229 | unclassified |  |  |
| Mute swan feces associated partitiviridae O | QUS52688.1 | *Durnavirales* | *Partitiviridae* | Unknow |
| Norway partiti-like virus 1 | ASY03273.1 | *Durnavirales* | *Partitiviridae* | Unknow |
| Lhasa Parti tick virus 1 | UYL95496.1 | *Durnavirales* | *Partitiviridae* | Unknow |

***Jingchuvirales***

| Lishi spider virus 1 | AJG39049.1 | *Jingchuvirales* | *Chuviridae* | *Chuvivirus* |
| --- | --- | --- | --- | --- |
| Lishi spider virus 1 | AJG39051.1 | *Jingchuvirales* | *Chuviridae* | *Chuvivirus* |
| Wenzhou crab virus 2 | AJG39061.1 | *Jingchuvirales* | *Chuviridae* | *Chuvivirus* |
| Hubei odonate virus 11 | APG78701.1 | *Jingchuvirales* | *Chuviridae* | *Odonatavirus* |
| Nasutitermes takasagoensis chuvirus 1 | DBA56554.1 | *Jingchuvirales* | *Chuviridae* | Unknow |
| Nasutitermes takasagoensis chuvirus 1 | DBA56558.1 | *Jingchuvirales* | *Chuviridae* | Unknow |
| Wuchang Cockroach Virus 3 | DBA56593.1 | *Jingchuvirales* | *Chuviridae* | *Scarabeuvirus* |
| Wuchang Cockroach Virus 3 | DBA56598.1 | *Jingchuvirales* | *Chuviridae* | *Scarabeuvirus* |
| megalopteran chu-related virus 119 | YP_010798609.1 | *Jingchuvirales* | *Crepuscuviridae* | *Aqualaruvirus* |
| Lampyris noctiluca chuvirus-like virus 1 | QBP37028.1 | *Jingchuvirales* | *Chuviridae* | *Scarabeuvirus* |
| Changping mivirus | QFR36195.1 | *Jingchuvirales* | *Chuviridae* | *Mivirus* |
| Coleopteran chu-related virus OKIAV151 | QMP82321.1 | *Jingchuvirales* | *Chuviridae* | Unknow |
| Hymenopteran chu-related virus OKIAV146 | QPL15382.1 | *Jingchuvirales* | *Chuviridae* | *Mivirus* |
| Neuropteran chu-related virus OKIAV150 | QPL15388.1 | *Jingchuvirales* | *Chuviridae* | Unknow |
| Orthopteran chu-related virus OKIAV152 | QPL15390.1 | *Jingchuvirales* | *Chuviridae* | Unknow |
| Culex mosquito virus 4 | QRW42853.1 | *Jingchuvirales* | *Chuviridae* | Unknow |
| Sanya chuvirus 1 | UHM27546.1 | *Jingchuvirales* | *Chuviridae* | Unknow |
| Hangzhou altica cyanea chuvirus 1 | UHR49731.1 | *Jingchuvirales* | *Chuviridae* | *Culicidavirus* |
| Nuomin virus | UKS70425.1 | *Jingchuvirales* | *Chuviridae* | *Chuvivirus* |
| Hebei mivirus 1 | URY50694.1 | *Jingchuvirales* | *Chuviridae* | *Chuvivirus* |
| Yanbian Chuvi tick virus 1 | UYL95469.1 | *Jingchuvirales* | *Chuviridae* | Unknow |
| Chuviridae sp | WAS28129.1 | *Jingchuvirales* | *Chuviridae* | Unknow |
| Wuhan mivirus | WAS28257.1 | *Jingchuvirales* | *Chuviridae* | *Mivirus* |
| Drosophila Burdiehouse burn chuvirus | WPV74286.1 | *Jingchuvirales* | *Chuviridae* | Unknow |
| Yichun mivirus | WWT48668.1 | *Jingchuvirales* | *Chuviridae* | Unknow |
| Aulacophora indica chu-like virus 1 | XIJ73171.1 | *Jingchuvirales* | *Chuviridae* | Unknow |
| Suffolk virus | YP_009177219.1 | *Jingchuvirales* | *Chuviridae* | *Mivirus* |
| Bole Tick Virus 3 | YP_009177702.1 | *Jingchuvirales* | *Chuviridae* | *Mivirus* |
| Tacheng Tick Virus 4 | YP_009177714.1 | *Jingchuvirales* | *Chuviridae* | *Mivirus* |
| Wuhan Mosquito Virus 8 | YP_009177720.1 | *Jingchuvirales* | *Chuviridae* | *Culicidavirus* |
| Imjin River virus 1 | YP_009182178.1 | *Jingchuvirales* | *Chuviridae* | *Mivirus* |
| Lonestar tick chuvirus 1 | YP_009254001.1 | *Jingchuvirales* | *Chuviridae* | *Mivirus* |
| Shayang Fly Virus 1 | YP_009300661.1 | *Jingchuvirales* | *Chuviridae* | *Morsusvirus* |
| Wenzhou Crab Virus 3 | YP_009302833.1 | *Jingchuvirales* | *Natareviridae* | *Charybdivirus* |
| Wenzhou Crab Virus 3 | YP_009302835.1 | *Jingchuvirales* | *Natareviridae* | *Charybdivirus* |
| Hubei myriapoda virus 8 | YP_009330110.1 | *Jingchuvirales* | *Chuviridae* | *Culicidavirus* |
| Beihai hermit crab virus 3 | YP_009333154.1 | *Jingchuvirales* | *Chuviridae* | *Mivirus* |
| Beihai barnacle virus 9 | YP_009333176.1 | *Jingchuvirales* | *Aliusviridae* | *Ollusvirus* |
| Wenling crustacean virus 15 | YP_009336633.1 | *Jingchuvirales* | *Natareviridae* | *Charybdivirus* |
| Hubei coleoptera virus 3 | YP_009336864.1 | *Jingchuvirales* | *Natareviridae* | *Charybdivirus* |
| Hubei chuvirus-like virus 3 | YP_009337089.1 | *Jingchuvirales* | *Myriaviridae* | *Myriavirus* |
| Hubei chuvirus-like virus 3 | YP_009337090.1 | *Jingchuvirales* | *Myriaviridae* | *Myriavirus* |
| Sanxia atyid shrimp virus 4 | YP_009337429.1 | *Jingchuvirales* | *Chuviridae* | *Pediavirus* |
| Wenling crustacean virus 14 | YP_009337857.1 | *Jingchuvirales* | *Chuviridae* | *Taceavirus* |
| Wenling crustacean virus 13 | YP_009337861.1 | *Jingchuvirales* | *Aliusviridae* | *Ollusvirus* |
| Hubei chuvirus-like virus 1 | YP_009337905.1 | *Jingchuvirales* | *Chuviridae* | *Scarabeuvirus* |
| Wuchang Cockroach Virus 3 | YP_009666257.1 | *Jingchuvirales* | *Chuviridae* | *Scarabeuvirus* |
| Culex mosquito virus 5 | YP_010797754.1 | *Jingchuvirales* | *Chuviridae* | *Piscichuvirus* |
| Lishi spider virus 1 | YP_010839349.1 | *Jingchuvirales* | *Chuviridae* | *Chuvivirus* |
