# Supplementary material for: Diversity and evolution analysis of RNA viruses in three wheat aphid species
Source: BMC Genomics. 2025 Apr 7;26:353. doi: 10.1186/s12864-025-11512-1 (PMC11978097; doi:10.1186/s12864-025-11512-1)
Supplement: Supplementary file 9 — Suppelemtary Material 9: Table S5. Identification of endogenous viral elements (EVEs) in the genomes of the three wheat aphids [file 12864_2025_11512_MOESM9_ESM.docx]

| **Table S5. Identification of endogenous viral elements (EVEs) in the genomes of the three wheat aphids^1,2^.** | | | | | | | |
| --- | --- | --- | --- | --- | --- | --- | --- |
| **EVE  names** | **Length** | **Chr** | **Chr position  (Orientation)** | **Homologous viruses** | **Protein hits** | **Identity** | **E-value** |
|  |  | **Scaffold** |  |  | **(aa position)** |  |  |
| SaChEVE1 | 568 | VSBQ01005201.1 | 1350-781(+) | Blacklegged tick chuvirus 2 | G(407-595) | 37.04% | 2.00E-36 |
| SaChEVE2 | 595 | VSBQ01000562.1 | 67016-66420(+) | Blacklegged tick chuvirus 2 | G(408-605) | 35.35% | 7.00E-37 |
| SaChEVE3 | 424 | VSBQ01000902.1 | 21741-21316(+) | Nuomin virus | G(402-525) | 37.90% | 1.00E-21 |
| SaChEVE4 | 544 | VSBQ01000051.1 | 902415-901870(+) | Orthopteran chu-related virus  OKIAV152 | G(414-596) | 31.69% | 5.00E-27 |
| SaChEVE5 | 547 | VSBQ01000289.1 | 10619-10071(-) | Suffolk virus | G(422-601) | 35.00% | 2.00E-34 |
| SaChEVE6 | 571 | VSBQ01000134.1 | 17988-17416(+) | Hymenopteran chu-related virus  OKIAV146 | G(404-591) | 37.23% | 1.00E-33 |
| SaChEVE7 | 574 | VSBQ01000731.1 | 15432-14857(+) | Wuhan Mosquito Virus 8 | G(401-585) | 33.68% | 1.00E-30 |
| SaChEVE8 | 604 | VSBQ01000906.1 | 16053-15448(-) | Hubei chuvirus-like virus 1 | G(408-603) | 36.68% | 1.00E-36 |
| SaChEVE9 | 577 | VSBQ01000731.1 | 12775-12197(+) | Belostoma flumineum  mononega-like virus | G(406-594) | 36.46% | 8.00E-33 |
| SaPaEVE1 | 645 | VSBQ01000304.1 | 248578-246036(-) | brine shrimp partitivirus 3 | RdRp(1-632) | 52.06% | 0 |
| SaPaEVE2 | 1285 | VSBQ01000464.1 | 23062-21776(-) | Hubei partiti-like virus 56 | RdRp(2-429) | 59.21% | 8E-179 |
| SaPaEVE3 | 1286 | VSBQ01000464.1 | 18377-17090(-) | Hubei partiti-like virus 56 | RdRp(2-429) | 59.21% | 8E-179 |
| SaPaEVE4 | 1290 | VSBQ01000464.1 | 27753-26462(-) | Hubei partiti-like virus 56 | RdRp(2-429) | 59.21% | 2E-176 |
| SgChEVE1 | 310 | CM037033.1 | 30049192-30048881(-) | Wuchang Cockroach Virus 3 | N(291-364) | 48.08% | 2.00E-20 |
| SgChEVE2 | 705 | CM037033.1 | 19755425-19754719(-) | Beetle chuvirus 2 | RdRp(514-745) | 56.84% | 2.00E-84 |
| SgChEVE3 | 552 | CM037033.1 | 19645008-19644455(-) | Beetle chuvirus 2 | RdRp(515-696) | 60.99% | 4.00E-71 |
| SgChEVE4 | 619 | CM037034.1 | 24661905-24661285(+) | Karukera tick virus | G(409-617) | 33.01% | 5.00E-25 |
| SgChEVE5 | 448 | CM037036.1 | 29039319-29038870(+) | Hebei mivirus 1 | G(463-607) | 30.34% | 3.00E-14 |
| SgChEVE6 | 529 | CM037034.1 | 71005755-71005225(+) | Blacklegged tick chuvirus 2 | G(417-591) | 39.55% | 2.00E-33 |
| SgChEVE7 | 542 | CM037036.1 | 24611506-24610963(+) | Blacklegged tick chuvirus 2 | G(417-595) | 40.20% | 6.00E-23 |
| SgChEVE8 | 595 | CM037036.1 | 2447919-2447323(-) | Blacklegged tick chuvirus 2 | G(408-605) | 35.35% | 2.00E-37 |
| SgChEVE9 | 867 | CM037033.1 | 35417382-35416514(+) | Blacklegged tick chuvirus 2 | G(314-605) | 37.65% | 9.00E-42 |
| SgChEVE10 | 547 | CM037031.1 | 17090610-17090062(-) | Hymenopteran chu-related  virus OKIAV146 | G(404-585) | 35.16% | 2.00E-34 |
| SgChEVE11 | 556 | CM037031.1 | 2644091-2643534(-) | Chuviridae sp. | G(3-187) | 34.05% | 4.00E-29 |
| SgChEVE12 | 565 | CM037031.1 | 61017715-61017149(-) | Hymenopteran chu-related virus OKIAV146 | G(403-590) | 37.23% | 1.00E-36 |
| SgChEVE13 | 890 | CM037036.1 | 6097007-6096116(-) | Hymenopteran chu-related virus  OKIAV146 | G(99-386) | 32.18% | 2.00E-30 |
| SgChEVE14 | 569 | CM037035.1 | 446825-446255(+) | Lampyris noctiluca chuvirus-like  virus 1 | E(408-596) | 38.64% | 2.00E-20 |
| SgChEVE15 | 574 | CM037033.1 | 24013805-24013230(-) | Orthopteran chu-related virus  OKIAV152 | G(406-596) | 37.50% | 6.00E-38 |
| SgChEVE16 | 575 | CM037033.1 | 24138126-24137550(-) | Coleopteran chu-related virus  OKIAV151 | G(432-620) | 29.53% | 9.00E-19 |
| SgChEVE17 | 595 | CM037033.1 | 24006634-24006038(-) | Orthopteran chu-related virus  OKIAV152 | G(407-603) | 33.67% | 3.00E-35 |
| SgChEVE18 | 598 | CM037035.1 | 12736545-12735946(-) | Lishi spider virus 1 | G(407-605) | 34.67% | 2.00E-26 |
| SgChEVE19 | 595 | CM037036.1 | 26515803-26515207(+) | Yanbian Chuvi tick virus 1 | G(408-605) | 34.85% | 2.00E-37 |
| SgChEVE20 | 615 | CM037031.1 | 88689389-88688773(+) | Lampyris noctiluca chuvirus-like virus 1 | E(408-611) | 33.00% | 2.00E-24 |
| SgChEVE21 | 1348 | CM037031.1 | 86971333-86969984(+) | Lampyris noctiluca chuvirus-like  virus 1 | E(139-592) | 28.90% | 3.00E-45 |
| SgChEVE22 | 1367 | CM037031.1 | 86980929-86979561(+) | Lampyris noctiluca chuvirus-like  virus 1 | E(139-592) | 32.75% | 5.00E-52 |
| SgChEVE23 | 1068 | CM037031.1 | 86975830-86974761(+) | Wuhan mivirus | G(133-491) | 32.80% | 2.00E-36 |
| SgChEVE24 | 1365 | CM037031.1 | 86976130-86974764(+) | Wuhan mivirus | G(132-588) | 26.84% | 4.00E-40 |
| SgChEVE25 | 1369 | CM037031.1 | 86864688-86863318(+) | Wuhan mivirus | G(132-588) | 28.25% | 4.00E-44 |
| SgChEVE26 | 1575 | CM037035.1 | 14263014-14261438(+) | Wuchang Cockroach Virus 3 | G(69-589) | 33.02% | 6.00E-99 |
| SgChEVE27 | 1575 | CM037036.1 | 13030893-13029317(+) | Wuchang Cockroach Virus 3 | G(77-593) | 35.63% | 1.00E-95 |
| SgChEVE28 | 1586 | CM037035.1 | 7271968-7270381(+) | Wuchang Cockroach Virus 3 | G(206-599) | 34.77% | 3.00E-83 |
| SgChEVE29 | 1591 | CM037031.1 | 10143173-10141581(+) | Hubei chuvirus-like virus 3 | G(84-616) | 31.09% | 5.00E-96 |
| SgChEVE30 | 1593 | CM037036.1 | 12632158-12630564(+) | Yichun mivirus | G(71-603) | 34.46% | 2.00E-106 |
| SgChEVE31 | 1593 | CM037036.1 | 17309752-17308158(+) | Yichun mivirus | G(71-603) | 34.64% | 2.00E-107 |
| SgChEVE32 | 1593 | CM037033.1 | 2291058-2289464(+) | Chuviridae sp. | G(71-603) | 34.46% | 2.00E-106 |
| SgChEVE33 | 1607 | CM037035.1 | 19287696-19286088(-) | Wuchang Cockroach Virus 3 | G(70-599) | 34.29% | 5.00E-77 |
| SgChEVE34 | 1764 | CM037033.1 | 47180167-47178402(-) | Yichun mivirus | G(21-605) | 32.49% | 7.00E-95 |
| SgMeEVE1 | 1702 | CM037031.1 | 60992393-60990690(+) | Ips erranti-like virus 2 | RdRp(798-1126) | 32.46% | 2.00E-25 |
| SgMeEVE2 | 2536 | CM037031.1 | 2648545-2646008(-) | Ips erranti-like virus 2 | RdRp(280-1126) | 35.70% | 2.00E-143 |
| SgMeEVE3 | 2104 | CM037033.1 | 65067364-65065259(+) | Ips erranti-like virus 5 | RdRp(280-1126) | 37.79% | 2.00E-162 |
| SgMeEVE4 | 6263 | CM037035.1 | 17853642-17847378(-) | Ips erranti-like virus 5 | RdRp(1-717) | 36.44% | 1.00E-106 |
| SgNyEVE1 | 315 | CM037033.1 | 30790355-30790039(-) | Beetle nyamivirus | RdRp(477-580) | 51.43% | 5.00E-27 |
| SgNyEVE2 | 1782 | CM037033.1 | 19735983-19734200(-) | Beetle nyamivirus | RdRp(157-764) | 43.59% | 4.00E-157 |
| SgNyEVE3 | 2094 | CM037033.1 | 40941605-40939510(+) | Beetle nyamivirus | RdRp(155-850) | 50.57% | 0 |
| SgNyEVE4 | 450 | CM037033.1 | 91913717-91913266(+) | Ixodes ricinus orinovirus-like virus 1 | RdRp(394-542) | 55.03% | 2.00E-47 |
| SgOrEVE1 | 472 | CM037033.1 | 19727547-19727074(-) | Blattodean orthomyxo-related virus  OKIAV181 | N(368-523) | 36.71% | 2.00E-21 |
| SgOrEVE2 | 523 | CM037033.1 | 14989476-14988952(-) | Old quarry swamp virus | N(234-399) | 40.80% | 2.00E-28 |
| SgOrEVE3 | 913 | CM037033.1 | 19602784-19601870(-) | Blattodean orthomyxo-related virus  OKIAV181 | N(228-523) | 35.74% | 2.00E-50 |
| SgOrEVE4 | 913 | CM037033.1 | 19607256-19606342(-) | Blattodean orthomyxo-related virus  OKIAV181 | N(228-523) | 35.74% | 2.00E-50 |
| SgOrEVE5 | 913 | CM037033.1 | 19611728-19610814(-) | Blattodean orthomyxo-related virus  OKIAV181 | N(228-523) | 35.74% | 2.00E-50 |
| SgOrEVE6 | 913 | CM037033.1 | 19616200-19615286(-) | Blattodean orthomyxo-related virus  OKIAV181 | N(228-523) | 35.74% | 2.00E-50 |
| SgOrEVE7 | 913 | CM037033.1 | 19694088-19693174(-) | Blattodean orthomyxo-related virus  OKIAV181 | N(228-523) | 35.74% | 4.00E-50 |
| SgRhEVE1 | 357 | CM037033.1 | 62087426-62087068(+) | Longquan Rhinolophus sinicus  vesiculovirus 1 | RdRp(534-647) | 63.48% | 3.00E-46 |
| SgRhEVE2 | 554 | CM037033.1 | 19716821-19716266(-) | Longquan Rhinolophus affinis  vesiculovirus 1 | RdRp(531-710) | 54.09% | 1.00E-50 |
| SgRhEVE3 | 507 | CM037033.1 | 62200991-62200483(-) | Longquan Rhinolophus sinicus  vesiculovirus 1 | RdRp(559-717) | 51.53% | 4.00E-38 |
| SgRhEVE4 | 579 | CM037033.1 | 19630147-19629567(-) | Vesicular stomatitis Indiana virus | RdRp(8-195) | 54.69% | 8.00E-68 |
| SgRhEVE5 | 579 | CM037033.1 | 19633212-19632632(-) | Vesicular stomatitis Indiana virus | RdRp(8-195) | 54.69% | 8.00E-68 |
| SgRhEVE6 | 579 | CM037033.1 | 19636276-19635696(-) | Vesicular stomatitis Indiana virus | RdRp(8-195) | 54.69% | 8.00E-68 |
| SgRhEVE7 | 3644 | CM037033.1 | 19627082-19623437(-) | Vesicular stomatitis Indiana virus | RdRp(8-195) | 54.69% | 2.00E-60 |
| SgRhEVE8 | 2355 | CM037033.1 | 11706140-11703784(+) | Soybean thrips rhabdo-like virus 1 | RdRp(231-963) | 44.60% | 8.00E-155 |
| SgRhEVE9 | 1990 | CM037033.1 | 62206824-62204833(-) | Wuhan Louse Fly Virus 10 | RdRp(287-480) | 29.13% | 5.00E-09 |
| SgRhEVE10 | 1030 | CM037033.1 | 62855972-62854941(-) | Soybean thrips rhabdo-like virus 1 | N(5-341) | 32.66% | 4.00E-52 |
| SgRhEVE11 | 1225 | CM037033.1 | 12828054-12826828(+) | Soybean thrips rhabdo-like virus 2 | N(28-417) | 30.75% | 3.00E-57 |
| SgToEVE1 | 937 | CM037031.1 | 10133655-10132717(+) | Sanya totivirus 12 | D(809-1122) | 39.87% | 6.00E-52 |
| SgToEVE2 | 937 | CM037031.1 | 10136890-10135952(+) | Sanya totivirus 12 | D(809-1122) | 39.87% | 4.00E-52 |
| SgToEVE3 | 1414 | CM037031.1 | 10130453-10129038(+) | Totiviridae sp. | CP(615-1085) | 37.76% | 2.00E-74 |
| SgToEVE4 | 4205 | CM037031.1 | 10127218-10123012(+) | Totiviridae sp. | CP(615-1085) | 37.50% | 2.00E-73 |
| SgPaEVE1 | 955 | CM037033.1 | 28645279-28644323(+) | Hubei partiti-like virus 56 | RdRp(168-449) | 55.48% | 2.00E-104 |
| RpChEVE1 | 565 | CM037071.1 | 18479800-18479234(-) | Hymenopteran chu-related virus OKIAV146 | G(403-590) | 35.64% | 1.00E-36 |
| RpChEVE2 | 1565 | CM037072.1 | 30451987-30450421(-) | Hymenopteran chu-related virus OKIAV146 | G(66-582) | 36.71% | 1.00E-78 |
| RpChEVE3 | 568 | CM037071.1 | 18778976-18778407(-) | Lampyris noctiluca chuvirus-like virus 1 | E(408-596) | 33.86% | 9.00E-35 |
| RpChEVE4 | 568 | CM037071.1 | 75179844-75179275(-) | Lampyris noctiluca chuvirus-like virus 1 | E(408-596) | 33.86% | 9.00E-35 |
| RpChEVE5 | 607 | CM037071.1 | 2046258-2045650(+) | Orthopteran chu-related virus OKIAV152 | G(405-603) | 36.45% | 3.00E-34 |
| RpChEVE6 | 698 | CM037070.1 | 7353081-7352382(+) | Sanya chuvirus 1 | G(170-395) | 34.78% | 1.00E-38 |
| RpChEVE7 | 1552 | CM037071.1 | 48834698-48833145(-) | Wuchang Cockroach Virus 3 | G(120-589) | 34.46% | 4.00E-96 |
| RpChEVE8 | 1584 | CM037071.1 | 72839117-72837532(+) | Yichun mivirus | G(71-598) | 34.85% | 3.00E-104 |
| RpChEVE9 | 1593 | CM037071.1 | 1751727-1750133(+) | Yichun mivirus | G(71-603) | 34.64% | 4.00E-107 |
| RpChEVE10 | 1593 | CM037071.1 | 74758227-74756633(-) | Yichun mivirus | G(70-603) | 34.77% | 1.00E-107 |
| RpPaEVE1 | 908 | CM037071.1 | 73138953-73138044(-) | Hubei partiti-like virus 56 | RdRp(7-308) | 61.22% | 4.00E-94 |
| RpPaEVE2 | 1140 | CM037071.1 | 60053869-60052728(+) | Hubei partiti-like virus 56 | RdRp(73-451) | 58.16% | 6.00E-158 |

1. Abbreviation: Chr, Chromosome;CP: Capsid protein; RdRP: RNA-dependent RNA polymerase; G: glycoprotein; N: Nucleocapsid protein; E: envelope protein; D: dehydrin; +: The orientation of EVE is in the same direction with the predicted amino acid of the viral protein; -: The orientation of EVE is reverse complemented with the predicted amino acid of the viral protein.
2. The sequences of EVEs are provided in a Source Data file.
